# Edible Mushroom Polysaccharides as Functional Food Ingredients for Metabolic Health: Preparation, Techno-Functional Behavior, and Requirements for Translation

**DOI:** 10.3390/foods15193514

**Published:** 2026-10-01

**Authors:** Ming Chen, Ruifan Zou, Jie Chen, Yuping Zhang, Huan Diao, Yayuan Xu

**Affiliations:** 1 Sericulture Research Institute, Anhui Academy of Agricultural Sciences, Hefei 230061, China; ming_ahas@foxmail.com (M.C.); zouruifan0805@126.com (R.Z.); ypzhang6330@163.com (Y.Z.); 2 Edible and Medicinal Mushroom Innovation Centre, Anhui Academy of Agricultural Sciences, Hefei 230061, China; 3 Institute of Agricultural Economics and Information, Anhui Academy of Agricultural Sciences, Hefei 230001, China; aaaskjwx@163.com; 4 Public Teaching Department, Discipline of Food Nutrition and Health, Anhui Finance & Trade Vocational College, Hefei 230601, China; 5 Institute of Agro-Products Processing, Anhui Academy of Agricultural Sciences, Hefei 230001, China

**Keywords:** edible mushroom polysaccharides, beta-glucans, functional food ingredients, metabolic health, techno-functional properties, dose translation, gut microbiota, rheology

## Abstract

Edible mushroom polysaccharide (EMP) preparations have repeatedly improved glycemic control, adiposity, lipid homeostasis and hepatic lipid accumulation in animal models, and luminal assays and microbiota-perturbation experiments support the routes involved. Selected preparations also thicken, emulsify, retain water and modify food matrices. Yet these findings rarely concern the same material: metabolic, mechanistic and techno-functional studies usually examine different preparations, and retained function is seldom assessed after formulation, processing and digestion. This narrative review uses preparation identity, defined by biological source, processing route and resulting material characteristics, to integrate preparation chemistry, gastrointestinal mechanisms, animal and human outcomes, food-system behavior and food-use considerations. Molecular size, solubility, hydration, binding and fermentability provide a shared physicochemical basis for food-matrix performance and gastrointestinal interactions. Animal doses, human intakes and food-matrix loadings overlap at gram scale, which leaves functional delivery as the unresolved step. Controlled human trials are still few, small and dissimilar in design, so translation remains to be tested. The central question is therefore whether a reproducible EMP preparation can retain its intended function in an acceptable food and produce matched human outcomes. Progress depends on following the same characterized preparation from production and food incorporation through processing, consumption and human evaluation.

## 1. Introduction

Global production of cultivated edible mushrooms has exceeded 40 million tonnes annually and is concentrated in a small number of leading genera [1,2]. Polysaccharides are a major macromolecular component of dried mushroom fruiting bodies [3,4], while powders, extracts and concentrates provide practical routes for incorporating this biomass into formulated foods [5]. Production scale and polysaccharide content provide a substantial raw-material basis for polysaccharide ingredients. Ingredient development, however, has not advanced at the same pace. Crude extracts, polysaccharide-enriched extracts, chromatographic fractions and purified polysaccharides represent different preparation classes, yet their composition, preparation history and application-specific performance are reported unevenly. The same broad label can encompass preparations that are difficult to compare or reproduce. Abundant biomass becomes useful for metabolic-health applications only when the resulting preparation can be recognized, reproduced and delivered with relevant functionality.

Edible mushroom polysaccharides (EMPs) are relevant to food development for metabolic health because their material properties connect formulation with processes in the gastrointestinal tract. Obesity, type 2 diabetes, dyslipidemia and hepatic steatosis share disturbances in insulin sensitivity, inflammatory regulation, bile-acid metabolism and gut microbial ecology [6,7]. Dietary macromolecules acting within the gastrointestinal tract can influence several of these processes through digestive, microbial and bile-acid routes [6,7,8,9]. For EMPs, hydration, binding and fermentability provide recurring points of overlap between food-system behavior and the gastrointestinal environment. Rodent studies have reported changes in glycemic control, adiposity, lipid homeostasis and hepatic lipid accumulation, while mechanistic work has used gut microbiota profiling, fecal microbiota transplantation, antibiotic depletion and measurements of digestive interactions or microbial metabolites [9]. Human interventions provide a more limited and heterogeneous picture. Differences in preparation type, dose, food vehicle and endpoint leave it uncertain whether preclinical responses can be reproduced with defined EMP preparations at food-relevant exposures [8].

Food-ingredient development also depends on performance in food systems. Research on mushroom-derived polysaccharides has examined their use as ingredients, matrix modifiers and processing-sensitive biomaterials [10,11,12,13,14,15,16]. Reported functions include fat replacement and changes in texture, cooking loss, hydration, viscosity, emulsification and protein-network behavior [11,12,13,14,15,16]; studies of films, coatings and hydrogels further show how processing and matrix interactions shape polysaccharide performance [10]. Yet the metabolic and food-system literature has largely advanced around different questions and, crucially, different preparations. Their species, material formats and endpoints rarely coincide. A preparation associated with a metabolic response may never be evaluated under food-processing conditions. Conversely, a technically promising ingredient may lack a corresponding exposure or metabolic assessment. The literature can therefore identify biological activity and technological functionality without showing that both belong to a preparation suitable for food use. Whether they converge in a reproducible preparation at a realistic intake remains a central translational question.

Earlier reviews have defined the major components of this problem. Recent syntheses have organized EMP research by extraction route, structural parameter, processing effect, anatomical site of action or an individual bioactivity [17,18,19,20,21]. Giavasis identified production consistency, interactions with food components, processing effects and human validation as important development issues [22], while Ritota and Manzi examined how *Pleurotus* powders and extracts affect product quality and sensory properties [23]. Together, these reviews clarify the components of EMP development, while their relationship remains unresolved at the level of the individual preparation. Species names, process labels and single structural descriptors cannot fully identify a functional material because extraction, purification and controlled degradation alter molecular distributions and co-extracted components; matrix conditions further shape behavior. The individual preparation therefore provides the relevant unit of comparison. Molecular size, solubility, hydration, binding and fermentability can influence food-system performance as well as events within the gastrointestinal tract. Accordingly, the present review uses preparation identity as the common basis for comparing metabolic responses, food-system behavior and exposure feasibility. Preparation identity is defined here by the biological source, processing route and resulting material characteristics. This perspective places the long-standing question of whether purified polysaccharides behave differently in the free state and in foods [24] within a direct food-ingredient development context.

This review evaluates EMP preparations as candidate functional food ingredients for metabolic-health applications. EMPs are defined here to include polysaccharides from cultivated culinary mushrooms and from widely consumed edible–medicinal fungi such as *Ganoderma* and *Cordyceps*. Eligibility for food use depends on the specific preparation and jurisdiction. Four practical questions organize the synthesis: what preparation is being used, how it behaves in a food matrix, what exposure it can realistically deliver and what metabolic outcome has been observed. Viscosity, solubility, molecular size, matrix entrapment and binding behavior can shape digestive access, bioaccessibility and glycemic or lipid responses [25,26,27,28]. Several of these features also influence hydration, thickening, emulsification and dispersion in formulated foods. The analysis centers on EMP preparations tested against metabolic outcomes and uses technological and regulatory findings to evaluate their suitability for food-ingredient development. Animal doses are considered alongside observed human intakes and food-matrix loadings. Quality, safety and regulatory status define additional conditions for translation. This preparation-centered synthesis and the practical recommendations proposed here offer a common analytical basis for future EMP studies.

The studies considered here were identified through structured searches of Web of Science and PubMed for English-language publications from 2021 to 2026. Search strings combined edible or medicinal mushroom names and polysaccharide terms with metabolic outcome terms (metabolic syndrome, type 2 diabetes, obesity, insulin resistance, dyslipidemia, fatty liver disease, atherosclerosis and glucose intolerance). Two hundred and twenty-two candidate publications were screened by title, abstract and full text, and 146 primary studies reporting a metabolic, mechanistic or food-relevant outcome for an identifiable EMP preparation were included. (The complete search strings, filters and eligibility criteria are given in the Supplementary Materials). Human trials, food-system and extraction studies, regulatory documents and reporting guidelines were added through targeted searches and reference lists. Controlled human studies were included when the tested product was a defined EMP preparation or a mushroom food format with a stated polysaccharide or β-glucan basis and a metabolic endpoint was reported; trials with non-metabolic primary endpoints and dietary-substitution studies of whole mushrooms without a stated polysaccharide basis were not included. The synthesis is narrative and thematic. Studies cited in the tables were selected as representative examples according to three criteria: a within-study comparison of preparations or conditions, numerical outcomes that could be verified in the original publication, and a preparation description sufficient to identify the source, processing route, and material class.

## 2. Preparation and Structural Features of EMPs

### 2.1. Fungal Sources and Material Categories

Across the studies examined in this review, metabolic and food-system research is distributed across partly different species and preparation formats. Metabolic investigations commonly include preparations from both culinary genera [29,30,31,32,33,34] and edible–medicinal fungi such as *Ganoderma* and *Cordyceps* [35,36,37,38,39,40]. By contrast, the food-matrix studies considered here more frequently involve *Auricularia*, *Tremella* and *Pleurotus* preparations [12,13,14,15,41,42,43,44,45,46,47]. When evaluating the translatability of a given finding to a food-ingredient context, species selection must be interpreted jointly with preparation format, biological endpoint and intended food application.

EMP preparations are obtained from fruiting bodies, mycelium and submerged-culture broth [17,19]. In the studies examined here, fruiting bodies are the most common starting material, whereas culture broth [48,49], spores [50] and processing residues [51,52,53] are used less frequently. Preparations derived from different parts of the same species can exhibit distinct properties: for example, fruiting-body and mycelial preparations of the same species may differ in polysaccharide content and monosaccharide composition [4,54] and in regulatory classification [55]. Commercially sourced preparations are uncommon in the metabolic studies covered in this review, whereas several food-system studies used commercial polysaccharides [14,41,42].

**Figure 1:**
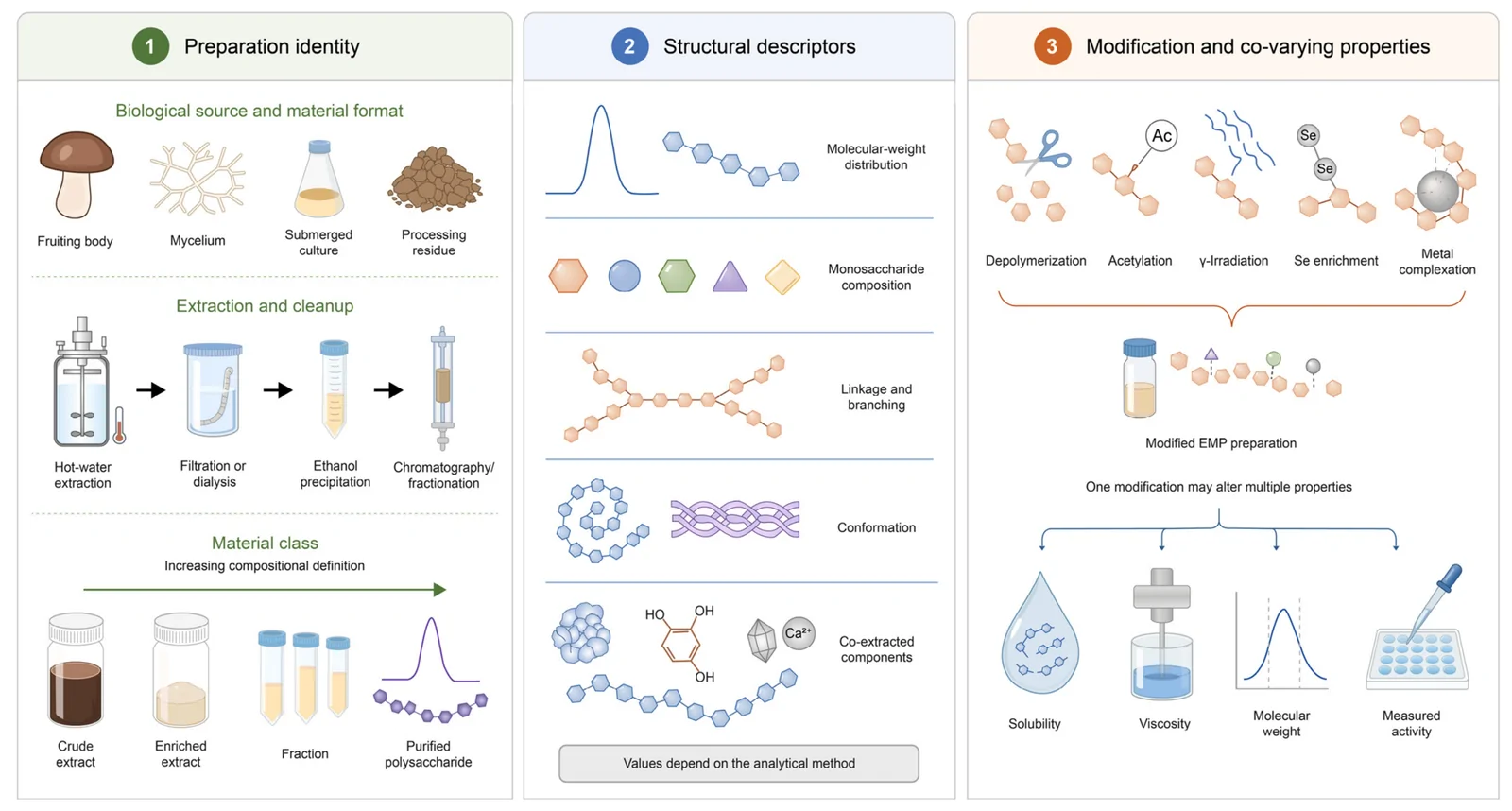
Preparation variables and structural descriptors that define an edible mushroom polysaccharide (EMP). (1) Biological source and material format, a representative hot-water extraction and cleanup sequence, and four material classes arranged by increasing compositional definition (crude extract, polysaccharide-enriched extract, chromatographic fraction and purified polysaccharide). (2) Structural descriptors of the resulting material: molecular-weight distribution, monosaccharide composition, linkage and branching, conformation and co-extracted components such as protein, phenolic compounds and salts. (3) Modification routes and the properties that a single modification can alter together (solubility, viscosity, molecular weight and measured activity). Ac, acetyl group; Se, selenium; gray sphere, complexed metal ion (Zn(II) or Cr(III)).

Based on their degree of compositional definition, EMP preparations can be grouped into four categories: crude extract, polysaccharide-enriched extract, chromatographic fraction and purified polysaccharide. This classification is based on the depth of preparation and separation reported in each study and does not rely on fixed purity thresholds. Enriched extracts are commonly represented in the selected studies [30,36,37,38,40,56,57,58] and are more compatible with ingredient-like formats than highly resolved fractions. Fractionated and purified materials are used primarily in mechanistic and structure–activity investigations [29,31,33,34,35,59], as they provide the clearest picture of which components have been removed. By contrast, bioactivity measured using an enriched extract remains attributable to the whole preparation rather than a single defined polymer. To illustrate the compositional differences across preparation formats, a food-application comparison reported an approximately two-fold difference in β-glucan content between dried *Pleurotus ostreatus* powder and the polysaccharide extract prepared from the same raw material [12]. Importantly, these categories describe application-relevant preparation formats and do not constitute a fixed hierarchy of quality. Figure 1 provides the organizing framework for these preparations by linking biological source and preparation class with structural descriptors and the co-varying effects of modification.

### 2.2. Extraction and Purification Routes

The extraction route used to isolate an EMP directly shapes the properties of the final material. Hot-water extraction followed by ethanol precipitation remains the benchmark reference route, while alkaline, enzymatic, ultrasonic and microwave-assisted methods are employed to modulate yield, molecular-weight distribution, chemical composition and measured bioactivity [17,19,60]. A recent comparative study of *Lentinula edodes* demonstrated that hot-water, hot-alkaline, ultrasound-water and ultrasound-alkaline extraction, each under optimized conditions, produced crude polysaccharides with differing yields, swelling behavior and enzyme-inhibition activity [56]. These comparisons confirm that processing conditions directly alter yield, structural features and measured activity, making the extraction protocol a key determinant of the final material rather than a neutral procedural step. Table 1 compares the main extraction and purification routes by their principle and effect on the polysaccharide, their advantages and limitations for ingredient production, and EMP examples.

Physical-field methods apply acoustic, electromagnetic, electrical or mechanical energy to the mushroom material, alone or in combination, to accelerate the release of polysaccharides into water [60]. Ultrasound acts mainly through acoustic cavitation: micro-jets, shock waves and shear forces rupture the cell wall and drive solvent into the tissue, which allows lower temperatures and shorter extraction. Microwaves disrupt hydrogen bonds through dipole rotation and ionic migration, which promotes solvent penetration and cell-wall disruption. Pulsed electric fields permeabilize cells by electroporation without heating, and high pressure or high-speed shearing opens the wall by mechanical force at low temperature [60]. Relative to hot-water extraction, ultrasound and microwaves shorten the process, reduce solvent use and usually raise yield; in the *L. edodes* comparison the ultrasound-water route gave the highest yield of the four routes [56]. Mechanical-field extraction has been studied least and gave lower yields than hot water in two species examined [60,61,62]. The energy that opens the cell wall also acts on the dissolved polymer. Excessive ultrasonic or microwave exposure degrades the polysaccharide, and ultrasound- and microwave-assisted extracts generally have a lower molecular weight than hot-water extracts of the same material [60]. For food use the consequences run in both directions. After field-assisted extraction or degradation, intrinsic viscosity and dynamic moduli decrease, solubility and thermal stability increase, and the effect on gelling differs between studies [60]. For *Auricularia polytricha*, the optimization of ultrasonic/microwave-assisted extraction was accordingly reported together with the rheology of the product [13]. Field type, power, time and temperature therefore belong to the description of a preparation, because one species extracted at different intensities can yield materials that differ in viscosity and gelling behavior.

Deep eutectic solvents (DESs) act through the solvent. They are mixtures of a hydrogen-bond acceptor and a hydrogen-bond donor that are liquid at the extraction temperature and dissolve cell-wall polysaccharides through hydrogen bonding [63,64,65]. Those composed of primary metabolites are termed natural DESs (NaDESs). Their combination with physical fields has been proposed as a next step for EMP extraction [60]. Three applications to fungal polysaccharides illustrate the approach. A temperature-responsive ethanolamine–o-cresol DES extracted 92.4 mg/g of *Ganoderma lucidum* polysaccharide at 60 °C, released 88.1% of it into the aqueous phase on cooling and still gave 81.8 mg/g in the fifth cycle [63]. The product had a lower molecular weight and a different monosaccharide profile from the water extract. For shiitake stalks, computational screening of 372 candidate NaDESs led to a carnitine–urea–water solvent that gave a triple-helix lentinan content of 14.7 g/100 g [64]. For black truffle, an ascomycete included here as a related fungal example, betaine–citric acid combined with ultrasound gave a polysaccharide yield of 9.38%, 1.4 times that of water [65]. The advantages are a higher recovery than water, a solvent composition that can be tuned to the target polymer and, in the first case, reuse of the solvent. The limitations concern the ingredient that results. DESs are viscous, so water is added and mass transfer remains slow. The solvent has to be separated from a polymer to which it binds, and its components range from betaine and citric acid, which are food ingredients, to ethanolamine and o-cresol, which are not. None of the three studies reported the residual solvent content of the recovered polysaccharide, and none examined viscosity, emulsification or behavior in a food matrix. The products were assessed by antioxidant or skin-related assays [63,65] or by triple-helix content alone [64]. For food use, the identity of the solvent and data on its removal become part of the preparation description.

**Table 1:** Extraction and purification routes for edible mushroom polysaccharides (EMPs).

Route	Principle and Effect on the Polysaccharide	Advantages	Limitations	EMP Example	Ref.
Hot-water extraction with ethanol precipitation	Diffusion of water-soluble glucans and heteropolysaccharides at near-boiling temperature; protein, phenolics and salts are co-extracted; high-Mw fractions are largely preserved	Food-grade solvents; simple equipment; reference route for comparison	Extraction over hours; high water and energy demand; wall-bound glucans are not recovered	*L. edodes* crude polysaccharide, the reference in a four-route comparison	[17,56]
Alkaline extraction	Alkali releases alkali-soluble, wall-bound β-glucans; chain degradation and loss of O-acetyl groups can occur	Access to water-insoluble glucans; higher recovery	Neutralization salts; structural alteration; effluent handling	*L. edodes* hot-alkaline route; *F. velutipes*, where alkali extraction gave a triple-helix β-glucan and water extraction an α-glucan-rich fraction	[56,66]
Enzyme-assisted extraction	Cell-wall-degrading enzymes release polysaccharides at moderate temperature and pH; enzyme protein can remain	Mild conditions; selectivity; low solvent demand	Enzyme cost; inactivation step; batch variability of enzyme preparations	*Tremella fuciformis*: adding ultrasound to enzymatic extraction lowered intrinsic viscosity	[17,67]
Ultrasound (acoustic field)	Cavitation ruptures the cell wall and drives solvent into the tissue; Mw and intrinsic viscosity decrease	Simple operation; short time; low temperature; higher yield	Long exposure degrades the polysaccharide; equipment cost	*L. edodes* ultrasound-water and ultrasound-alkaline routes	[56,60]
Microwave (electromagnetic field)	Dipole rotation and ionic migration disrupt hydrogen bonds; rapid heating; Mw decreases	Low solvent use; short time; good reproducibility	Uneven heating; energy consumption; degradation at long exposure	*Auricularia auricula*: microwave-assisted extract with higher solubility and lower Mw	[60,68]
Combined ultrasound–microwave	Cavitation and rapid heating applied together or in sequence	Higher yield and shorter time than either field alone	Two sets of process parameters to control and report	Ultrasonic/microwave-assisted extraction (UMAE) of *A. polytricha*, reported with product rheology	[13,60]
Pulsed electric field	Electroporation by short high-voltage pulses; non-thermal	Continuous operation; short time; suited to heat-sensitive polysaccharides	Energy consumption; long treatment damages polymer conformation	*A. auricula* polysaccharide, compared with microwave and ultrasound extraction	[60,69]
Mechanical field (high pressure, high-speed shearing)	Pressure or shear force opens the wall at low temperature; lower-Mw products reported	Short time; low solvent use; few by-products	Equipment cost; filtration or centrifugation required; yields below hot water in the two species examined	*Schizophyllum commune* and *Clitocybe squamulosa* (high pressure); *Agaricus blazei* α-glucan (high-speed shearing)	[60,61,62,70]
Deep eutectic solvents (DES, NaDES)	Hydrogen-bond acceptor–donor mixtures dissolve wall polysaccharides; the product can differ from the water extract in Mw and monosaccharide profile	Higher recovery than water; tunable composition; solvent reuse shown for a temperature-responsive system	Viscous solvent; separation from the polymer; food compatibility of the components varies; residual solvent and food-system behavior not yet reported	*G. lucidum* (ethanolamine–o-cresol); shiitake stalks (carnitine–urea); black truffle (betaine–citric acid with ultrasound)	[63,64,65]
Purification (graded ethanol precipitation, deproteinization, membrane and column steps)	Compositional definition increases; protein, phenolics and salts are removed	Clear structure–activity attribution; batch consistency	Lower yield; cost; loss of the techno-functional contribution of co-extracted components	*L. edodes* graded ethanol fractions; *C. cicadae* column fractions	[35,59]

Notes: Routes were selected to cover the extraction principles applied to EMPs in the cited studies; the examples are studies that reported the route together with yield or material characterization. Black truffle is an ascomycete outside the cultivated mushrooms defined in the Introduction. DES, deep eutectic solvent; NaDES, natural deep eutectic solvent; Mw, weight-average molecular weight; UMAE, ultrasonic/microwave-assisted extraction.

Reported yield values correspond to different denominators and different stages of the preparation process, making a direct cross-study comparison of raw numerical values misleading. The three DES studies above express yield as crude polysaccharide per gram of raw material, as triple-helix β-glucan per 100 g of dry stalk and as percent crude polysaccharide [63,64,65]. In the *Lentinula edodes* comparison noted above, optimized ultrasound-water extraction achieved a yield of 35.2%, compared with 12.9% for hot-water extraction [56]. A study of *Auricularia auricula-judae* reported a crude extraction yield of 13.5%, alongside separate recovery values for subsequent purification and degradation steps [29]. In *Cordyceps cicadae*, by contrast, reported values of 19.3%, 46.2% and 34.3% described the distribution of fractions after column separation, not the extraction yield from starting raw material [35]. Yield data are most informative when interpreted in the context of their denominator and corresponding process stage; a low numerical yield does not inherently indicate an inefficient process.

Extraction conditions reported in the literature have generally been optimized for yield, compositional profile or measured bioactivity, reflecting the primary objectives of the underlying studies. Head-to-head comparisons of these conditions demonstrate how controllable processing choices shape the properties of the resulting EMP preparation and provide a basis for selecting candidate routes for further development. Translating these laboratory protocols to commercial ingredient manufacture requires additional assessment of process-level variables including energy demand, solvent recovery, batch scale and processing cost [60]. These practical factors bridge the gap between laboratory optimization and manufacturing feasibility.

Purification depth introduces a distinct set of trade-offs. Deeper purification improves compositional definition and simplifies structure–activity attribution, but it can also alter yield, solubility, emulsifying capacity and other properties relevant to food-matrix performance [59], while increasing processing complexity and cost. Purification is therefore best understood as a continuous preparation variable, not a simple gradient of increasing quality: the material that enables the clearest compositional attribution of bioactivity is not necessarily the material best suited for practical food-ingredient applications.

### 2.3. Structural Characteristics

When EMPs are evaluated as food ingredients, their structural characteristics provide an analytical basis for relating preparation conditions to physicochemical behavior and measured biological activity. Molecular-weight distribution is a particularly important structural descriptor, as reported EMPs span an extremely wide range from sub-kilodalton fractions to multi-megadalton polysaccharides [18]. This broad distribution reflects substantial structural diversity across EMP preparations and helps explain observed differences in their functional performance. It is important to note that molecular-weight estimates depend on the analytical method used: values obtained via size-exclusion or gel-permeation chromatography calibrated against dextran standards, size-exclusion chromatography coupled with multi-angle light scattering, and intrinsic-viscosity estimation are not directly interchangeable [4,18,71]. Molecular-weight data are most reliably interpreted within the measurement context in which they were generated.

Glucan content and monosaccharide composition provide additional compositional resolution beyond molecular-weight distribution. Glucan values measured by enzymatic, hydrolytic or spectrophotometric methods reflect different analytical definitions and are most appropriately compared across studies that use a common method [4]. Monosaccharide profiling further distinguishes glucose-rich glucans from heteropolysaccharides containing varying proportions of mannose, galactose, xylose, fucose and uronic acids [4,18]. Like molecular weight, the interpretation of monosaccharide profiles depends on the specific hydrolysis, detection and normalization procedures employed. Molecular-weight and compositional data describe distinct but complementary dimensions of EMP structure.

Glycosidic-linkage patterns, branching architecture and solution conformation provide a deeper level of structural resolution. Selected EMP preparations have been shown to adopt triple-helical conformations [4,18], and linkage/branching analysis can distinguish between materials that have otherwise similar molecular-weight and monosaccharide profiles. The term “purity” describes another dimension of preparation properties, but it is not a uniformly defined metric: it may refer to total sugar content, total polysaccharide content or chromatographic homogeneity. These different measures should each be retained alongside their specific analytical definitions and do not form a single, universal quality scale. Combining composition, molecular-weight distribution, linkage type, branching, conformation and co-extracted components provides a layered, multi-dimensional description of EMP properties that can be correlated with subsequent processing behavior and functional performance.

While broader relationships between extraction conditions, structural features and bioactivity have been reviewed previously [19], this section examines which manipulated preparation variables show consistent associations with measured metabolic outcomes. Four paired comparison studies demonstrate a recurring association between downward shifts in molecular weight and stronger measured activity. First, controlled degradation of *Auricularia auricula-judae* polysaccharide reduced its molecular weight from 119 to 59 kDa and decreased its α-glucosidase IC50 from 2.24 to 1.39 mg/mL [29]. Second, steam explosion treatment of *Flammulina velutipes* soluble dietary fiber reduced its molecular weight from 313 to 123 kDa and increased triglyceride suppression in oleic-acid-treated HepG2 cells from 28.4% to 51.8% at a dose of 800 μg/mL [51]. Third, γ irradiation of *Auricularia polytricha* polysaccharide reduced its molecular weight from 6820 to 34 kDa and lowered serum total cholesterol in mice from 5.93 to 4.37 mmol/L [30]. Fourth, ultrasound-assisted enzymatic treatment of *Hericium erinaceus* dietary fiber shifted its molecular-weight distribution downward, reduced its D50 particle size from 314 to 177 μm and increased pancreatic-lipase inhibition from 52.4% to 81.7% [52]. In each pair, the lower-molecular-weight preparation gave the stronger response within its own assay system.

These findings must be interpreted with appropriate caveats. The four studies used two enzyme assays, one cell model and one mouse model, so the magnitudes of their effects cannot be directly compared. In addition, each treatment altered multiple properties beyond molecular weight, including particle size, composition, viscosity and other chemical features. Because none of the comparisons varied molecular weight with these properties held constant, they do not establish molecular weight as an independent predictor of activity. Nevertheless, molecular weight remains an important variable. Its change records what a process has done to the polymer, and in oat β-glucan the cholesterol-lowering effect has been related to viscosity, to which molecular weight contributes [27]. Molecular-weight reduction is therefore best regarded as a recurring co-varying descriptor of enhanced measured activity in these paired studies. It is most informative within a preparation series and of limited value for ranking preparations from different sources or routes. Notably, the γ-irradiation study co-reported viscosity and animal metabolic outcomes for the same preparation series, illustrating how structural, physical and biological responses can be jointly characterized within a single set of modified materials.

### 2.4. Modification and Controlled Degradation

Modification extends the scope of preparation control beyond extraction and purification by deliberately altering polymer chain length, substituent groups or associated elements. Among the EMP studies considered here, modification approaches linked to metabolic outcomes fall into three broad classes: controlled degradation, chemical substitution, and elemental enrichment, complexation, or formulation. Controlled degradation encompasses chemical, physical and enzyme-assisted routes, represented here by peroxide-ascorbate treatment, steam explosion, ultrasound-assisted enzymatic treatment and γ irradiation [29,30,51,52]. Within-study substitution contrasts are provided by deacetylation [57] and acetylation [36], while selenium enrichment, polysaccharide-stabilized selenium nanoparticles, and Zn(II) or Cr(III) chelation represent approaches that modify elemental composition or formulation [37,38,50,72]. Because these routes differ in their chemical targets and treatment intensity, the modified material itself—rather than the route label alone—is the appropriate unit for comparison.

Each class of modification is characterized by route-specific descriptive metrics. Degree of substitution (DS) was used to describe acetyl-group content in the *Auricularia* and *Ganoderma* substitution studies [36,57], while element content served as the corresponding metric for selenium enrichment [37,73]. Post-modification molecular-weight distribution and compositional analysis can indicate whether the intended treatment was accompanied by unintended depolymerization or broader structural change. These descriptors support comparisons within a given modification class, although no single scalar measure is universally applicable across substitution, enrichment, chelation and nanoparticle formulation approaches. Sulfation and carboxymethylation, common modification routes for plant and cereal polysaccharides [74], are not represented in the metabolic EMP literature covered here.

The magnitude and nature of modification effects depend on both the specific route and the study comparison design. Controlled degradation showed the most consistent enhancement of measured activity across the paired in vitro and cell-based comparisons [29,51,52]. Acetylation altered multiple outcomes relevant to type 2 diabetes [36], while Zn(II) and Cr(III) complexation were associated with stronger enzyme inhibition or metabolic responses in their respective within-study comparisons [38,72]. Selenium enrichment followed a different pattern: at a matched dose of 100 mg/kg, selenium-rich *Cordyceps militaris* polysaccharide showed no clear advantage over the unenriched preparation, and the stronger response observed at 200 mg/kg cannot be attributed to selenium enrichment independently of dose [37]. Modification does not produce a uniform activity gain across routes; effects are specific to the treatment and experimental design.

When a modified EMP is incorporated into a food matrix, interactions with proteins, polyphenols and other components can further alter its conformation and rheological behavior [75]. For food-ingredient translation, the relevant evaluation is the modified preparation’s full structural, physical and safety profile at a defined application dose; assessment based solely on activity endpoint data is insufficient. Representative paired contrasts across extraction, degradation and other modification routes are summarized in Table 2, linking process variables with material changes, measured responses and food-ingredient implications.

## 3. Metabolic Effects and Mechanisms

**Figure 2:**
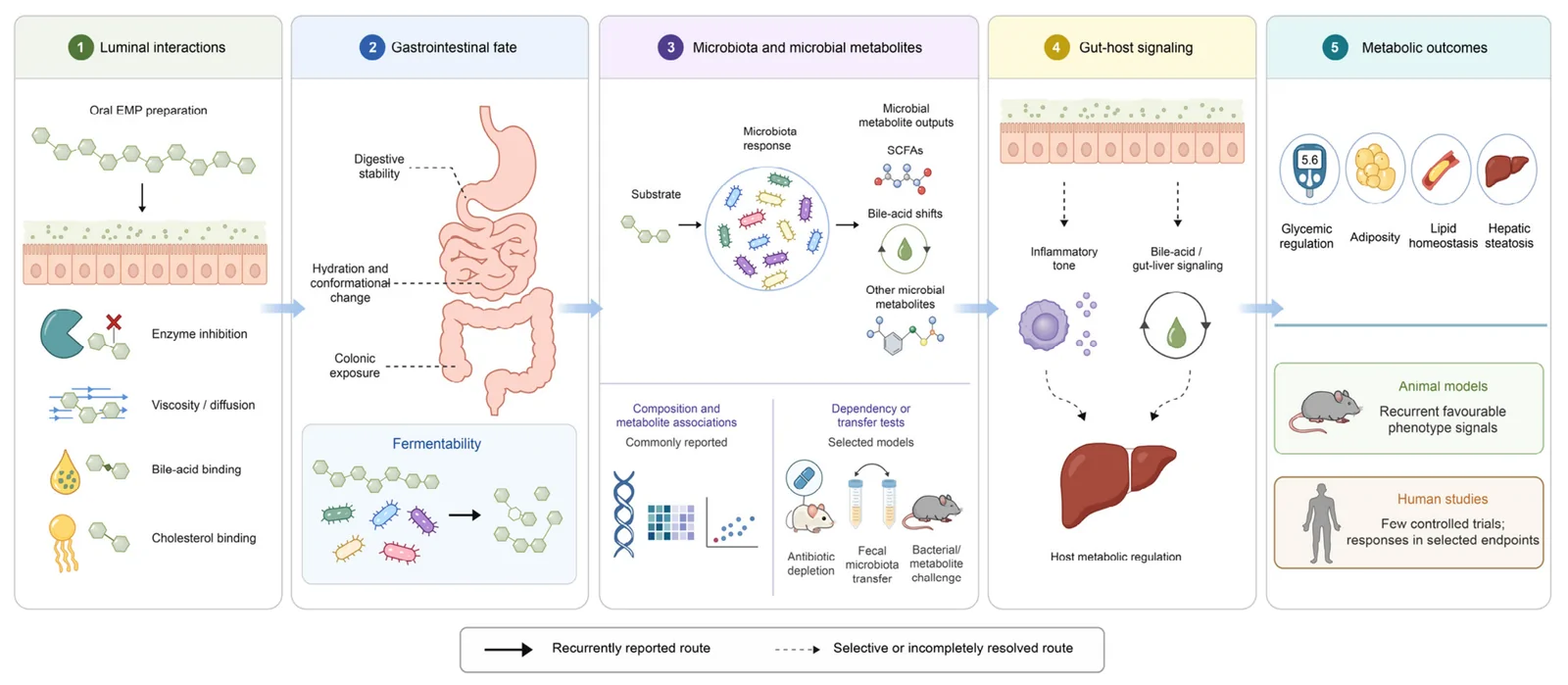
Biological-response routes and metabolic outcomes reported for orally administered edible mushroom polysaccharides (EMPs). The map follows an ingested preparation through (1) luminal interactions, (2) gastrointestinal fate, (3) the microbiota response and its metabolite outputs, including short-chain fatty acids (SCFAs), (4) gut–host signaling and (5) metabolic outcomes.

EMP studies encompass markedly different preparation formats. Molecular-size distribution, composition and processing history can change luminal behavior and coincide with different biological responses; consequently, findings cannot be transferred automatically between materials [29,31,52]. Animal studies provide the broadest phenotype data. Two gut-centered routes then organize the mechanistic literature: physicochemical interactions within the gastrointestinal lumen and microbiota-dependent metabolic signaling. Human trials test whether responses remain detectable in defined populations and food-delivery formats. Figure 2 provides the organizing map for these studies, moving from luminal interactions and gastrointestinal fate through microbiota-related routes to host metabolic outcomes, while distinguishing recurrently reported from selectively resolved links.

### 3.1. Preclinical Outcomes

Animal studies report a recurrent pattern across four connected metabolic domains: glycemic control, adiposity, lipid homeostasis and hepatic lipid accumulation. In diabetic models, *Auricularia* and *Cordyceps* preparations lowered fasting glucose and improved glucose-tolerance measures [39,58]. *Auricularia* polysaccharides also attenuated weight gain and improved glycemic, lipid and inflammatory profiles in high-fat-diet models [32,76]. At the gut–liver interface, *Cordyceps guangdongensis* and *Grifola frondosa* preparations were associated with changes in hepatic lipid metabolism, bile-acid regulation and related systemic outcomes [33,40]. Together, these studies establish a broad preclinical phenotype pattern across selected EMP preparations.

Preparation-related variation can be examined most directly in studies that compare materials within the same experimental design. Controlled degradation of an *Auricularia auricula-judae* polysaccharide preparation (AAP-1) reduced its molecular weight and changed its monosaccharide ratios and surface morphology. At a matched dose, the resulting degraded preparation (DAAP-1) showed stronger enzyme inhibition and more pronounced glycemic and lipid-related responses than native AAP-1 [29]. A fraction-level comparison in *Pleurotus citrinopileatus* produced a different molecular-weight pattern: the 1000 kDa fraction elicited stronger lipid-related responses than the 10 kDa fraction at the same dose and also showed greater fat binding and pancreatic-lipase inhibition [31]. In a head-to-head comparison of polysaccharides from five edible fungi, the *Agaricus bisporus* preparation produced the strongest antidiabetic response under the study conditions [34].

The degradation and fractionation contrasts vary the material within one species at a matched dose, so the changed response can be attributed to the changed preparation [29,31]. Their direction differed: lower molecular weight strengthened the response in one series and higher molecular weight in the other. The five-fungus comparison varies source and preparation together and cannot show which attribute was responsible [34]. Because molecular weight also co-varied with composition, morphology or fraction identity, it is not an independent or transferable predictor of metabolic activity.

These phenotypes raise a focused mechanistic question: how can preparation-dependent behavior in the gut contribute to systemic metabolic responses? Direct interactions with digestive components can alter nutrient handling, whereas microbial fermentation and community responses can change the metabolites presented to the host. These routes are experimentally distinct and biologically connected.

### 3.2. Luminal Mechanisms

Three processes recur within the gastrointestinal lumen: digestive-enzyme inhibition, sterol and bile-acid binding, and colonic fermentation. Hydration, viscosity and conformational behavior influence how polysaccharide-containing preparations interact with digestive components and remain available to the colonic microbiota [27,28]. Enzyme inhibition and binding represent direct physicochemical interactions under the assay conditions. Fermentation marks the transition from substrate behavior to microbial metabolism.

Digestive-enzyme inhibition provides the most detailed matched comparisons of processing and luminal activity. In the paired *Auricularia* degradation study, DAAP-1 lowered the α-glucosidase half-maximal inhibitory concentration (IC50) from 2.24 to 1.39 mg/mL and the α-amylase IC50 from 2.63 to 1.66 mg/mL relative to native AAP-1 [29]. The same preparation pair was tested in vitro and in mice, allowing the assay and in vivo contrasts to be interpreted together [29]. Similarly, four extraction routes applied to *Lentinula edodes* produced α-glucosidase IC50 values of 1.21–2.49 mg/mL and α-amylase IC50 values of 1.46–1.85 mg/mL [56]. Ultrasound-assisted enzymatic treatment of *Hericium erinaceus* dietary fiber increased pancreatic-lipase inhibition from 52.4% to 81.7% [52]. Processing changed both the preparation and its assay response in each comparison. The analytical value lies in the within-study contrast, since enzyme sources, substrates and assay conditions differ across papers.

Sterol and bile-acid binding defines a second luminal route relevant to intestinal lipid handling. A processed *Hericium erinaceus* dietary-fiber preparation showed higher cholesterol binding at pH 7 and higher glycocholate and taurocholate binding than the parent fiber in vitro [52]. In *Lentinula edodes*, a purified polysaccharide subfraction tested at 1 mg/mL bound 64.4 mg/g cholesterol and reached 91.9% of the cholate-binding capacity of cholestyramine [59]. These assays quantify binding capacity under defined conditions and provide a physicochemical basis for altered sterol and bile-acid availability in the intestinal lumen. The magnitude remains specific to the tested preparation and assay system.

Colonic fermentation carries preparation-specific substrate behavior into microbial metabolism. During in vitro fermentation of a *Dictyophora indusiata* polysaccharide, total short-chain fatty acids (SCFAs) increased from 7.7 mmol/L at 12 h to 16.0 mmol/L at 48 h [77]. A high-molecular-weight *Lentinula edodes* polysaccharide generated 80 mmol/L acetate, propionate and *n*-butyrate after 48 h of simulated human fermentation, compared with 39 mmol/L in the blank control and 103 mmol/L with fructo-oligosaccharide [78]. A *Pleurotus eryngii* polysaccharide likewise retained its molecular-weight profile during simulated upper-gastrointestinal digestion and was subsequently utilized during fecal fermentation, accompanied by SCFA production [79]. Consistent with these fermentation data, restoration of SCFA concentrations accompanied metabolic improvement in *Cordyceps cicadae*-treated diabetic mice [80] and *Trametes versicolor*-treated high-fat-diet mice [48]. In contrast, selenium-rich *Cordyceps militaris* polysaccharides improved lipid-related outcomes while fecal SCFA concentrations remained stable [37]. SCFA production is therefore one recurrent route within a broader microbial metabolic response.

### 3.3. Microbiota-Dependent Mechanisms

Microbiota profiling reveals repeated co-variation between EMP administration, community structure and metabolic phenotype. Across diabetic, obesity and gut–liver models, studies have reported changes in diversity, selected taxa, SCFAs, bile acids and other fecal metabolites alongside metabolic improvement [9,32,39,40,48,76,80]. Such coordinated patterns identify candidate microbial links and define hypotheses for perturbation experiments. Taxonomic abundance alone provides limited information about the functional contribution of an organism; metabolite measurements and intervention designs add the required biological context.

Perturbation designs provide stronger grounds for inferring microbial participation than taxonomic association alone [81,82]. In EMP research, this approach has been applied in two *Auricularia*-based obesity studies, where fecal microbiota transplantation (FMT) from treated donors transferred part of the metabolic phenotype to recipient mice [32,76]. Antibiotic depletion and *Papillibacter cinnamivorans* gavage further showed that a defined microbial intervention could reproduce part of the response to the tested *Auricularia* polysaccharide in the model [76]. A *Cordyceps cicadae* study reported two complementary experiments: FMT from polysaccharide-treated donors improved glycemic outcomes in recipient mice, and a separate indole intervention improved hyperglycemia and insulin sensitivity [39]. At the gut–liver interface, fecal transfer reproduced part of the hepatoprotective response associated with *Hericium erinaceus* polysaccharide treatment in aged laying hens [83], and microbiota depletion plus transfer linked *Auricularia* polysaccharide treatment with hepatic fibrosis outcomes in mice [84]. The evidential reach of each design is specific: FMT tests transferability of a treated-donor community, depletion tests dependence under disrupted microbial ecology, and organism or metabolite interventions localize selected links.

The most coherent synthesis is a family of preparation-specific microbial routes. Preparation identity, dose, dietary background, disease model and baseline community can each shape the observed response. Distinct community configurations may consequently converge on related glycemic, lipid or gut–liver outcomes through different combinations of microbial metabolites and host signaling pathways.

### 3.4. Human Studies

Human studies follow a different pattern and remain few. Only five controlled trials were identified, two testing defined preparations and three testing mushroom-based food formats [85,86,87,88,89]. These trials have produced measurable responses in selected endpoints, with limited replication across studies. Material format, participant phenotype, intervention duration and endpoint selection vary simultaneously; these design differences define the current translational boundary.

Trials of defined preparations have evaluated different biological domains. In 60 obese adults with cardiometabolic syndrome, 750 mg/day *Ganoderma lucidum* polysaccharide peptide (540 mg/day β-glucan) for eight weeks produced no between-group differences in the primary inflammatory endpoints, interleukin-6 (IL-6), tumor necrosis factor-α (TNF-α) and high-sensitivity C-reactive protein (hsCRP), or in nitric oxide, lipid profile and body mass index (BMI) [85]. A separate trial randomized 57 adults (52 analyzed) with untreated mild hypercholesterolemia and delivered a β-glucan-enriched *Lentinula edodes* mixture in commercial vegetable creams, providing 3.5 g/day fungal β-glucans for eight weeks. Microbiota composition changed, whereas total cholesterol, the registered primary endpoint, low-density lipoprotein cholesterol (LDL-C; +0.08 mmol/L with the mixture and +0.01 mmol/L with placebo) and inflammatory endpoints remained similar to placebo [86]. Only the *Lentinula* trial directly assessed microbiota composition. Across the two studies, endpoint selection determined which biological responses could be detected and compared.

Food-format trials extend the translation question to complex mushroom matrices. In an acute crossover trial of 22 adults with impaired glucose tolerance, a meal fortified with 20 g of oven-dried *Pleurotus ostreatus* powder provided 8.1 g of β-glucans and increased the viscosity of the test smoothie and soup by 142% and 53% at 37 °C. Postprandial glucose area under the curve (AUC), the primary endpoint, remained unchanged (paired difference +234 mg/dL·min; 95% confidence interval (CI) −225 to 704). At prespecified secondary endpoints, glucagon-like peptide-1 (GLP-1) AUC increased by 17% (766 pmol/L·min; 338 to 1190), non-esterified fatty acid AUC decreased by 14% and hunger AUC decreased by 22% (−1460 mm·min; −2680 to −282) [87]. A later randomized trial supplied 8.4 g/day *Pleurotus ostreatus* powder, equivalent to 3 g/day β-glucans, in a beverage to 50 adults, 46 of whom were analyzed, for four weeks. LDL-C, the primary endpoint, did not differ between groups (difference +0.12 mmol/L; 95% CI −0.14 to 0.34), other major lipid measures remained stable, and post hoc sex-adjusted analyses identified changes in cholesterol-absorption markers [88]. These trials place endocrine, lipid-flux and absorption markers among the early human responses to *Pleurotus* powder. The intervention was whole mushroom powder, so EMP-specific attribution remains unresolved.

A 12-week exploratory trial in 56 overweight or obese adults with prediabetes used a once-daily 180 mL *Tremella fuciformis* beverage declared to supply 6.4 g of β-glucan per serving, with anthropometric, metabolic and biochemical measures all listed as primary endpoints, and reported within-group reductions in HbA1c (6.03% to 5.96%) and waist circumference (95.2 to 93.5 cm), with fasting and post-load glucose, insulin-sensitivity indices and lipids unchanged and no between-group comparison reported [89]. The beverage was a boiled whole-mushroom extract; so, as for the *Pleurotus* trials, attribution to a defined EMP preparation remains unresolved.

The human findings show structured heterogeneity. Defined preparations, food matrices, participant characteristics and measured endpoints differ across trials, and conventional glycemic, lipid and inflammatory outcomes have not shown a consistent pattern. The trials delivered 0.54–8.1 g of declared β-glucan per day or per meal, lasted from a single meal to 12 weeks and analyzed 22–60 participants. Their primary endpoints were inflammatory in one trial, lipid-related in two and glycemic in one, and the fifth listed all measures as primary. The two trials of adults with elevated cholesterol at about 3 g/day β-glucan are the most comparable pair: LDL-C was the main comparison endpoint, total cholesterol, high-density lipoprotein cholesterol and triglycerides were also measured, and none differed between groups [86,88]. The responses observed so far come from secondary, post hoc or within-group analyses: GLP-1, hunger, and non-esterified fatty acids after one meal [87], cholesterol-absorption markers [88], and HbA1c and waist circumference [89]. They are early signals that require confirmation in trials designed for them. The current designs also leave the sources of between-study variation unresolved because processing, matrix behavior, luminal function and host phenotype were not separated within the same comparison.

Beyond the design issues above, sample size imposes a more fundamental limitation. Four of the five trials used a parallel-group design and enrolled 46–60 participants, roughly 23–30 per arm [85,86,88,89]; the fifth was an acute crossover trial in 22 participants [87]. The power of such designs can be estimated from the reported group sizes with a standard t-test at a two-sided significance level of 0.05. A parallel-group trial of this size reaches 80% power only when the true difference between groups is about 0.75–0.85 standard deviations. If the true difference is 0.5 standard deviations, power falls below 50%, and at 0.3 standard deviations it is approximately 20%. The crossover trial gains statistical efficiency from within-subject control, yet it reaches 80% power only for standardized within-person differences of about 0.6, and it addresses postprandial endpoints alone. Under these conditions, most trials would return non-significant results, and findings would vary widely across studies, even if EMPs exert a true effect.

The lack of a consistent pattern is therefore the expected outcome of a small, underpowered and heterogeneous set of trials, whether or not an underlying effect exists. An absence of effect cannot be inferred from it. A decisive study would be adequately powered and characterize the delivered preparation, its food-matrix and luminal behavior, baseline microbiota and host endpoints within the same intervention. Table 3 compares representative animal and human studies by preparation, exposure, design and principal outcome. Across in vitro, animal and human studies, hydration, viscosity, binding and fermentability operate at both biological and food-system interfaces. Their dual role makes retained functionality in food matrices the next translational question.

## 4. Techno-Functional Behavior in Food Systems

Techno-functional behavior determines whether an edible mushroom polysaccharide (EMP) preparation can be dispersed, stabilized, texturized and delivered at a feasible level in food. Its performance depends on the preparation’s structural and compositional features, the surrounding food matrix and the test conditions. A property label such as “soluble”, “viscous” or “emulsifying” has limited meaning unless concentration, pH, temperature, shear, composition and storage history are also known. This review distinguishes two kinds of functionality by where the measurement is made. Technological functionality is the performance of the preparation in a food under formulation, processing and storage conditions; health functionality is a physiological or metabolic response measured in the host after ingestion. Measurements made outside the food and the host belong to neither: they comprise properties of the preparation, such as viscosity, binding capacity and fermentability, and in vitro activity, such as enzyme inhibition or the responses of cultured cells. A property can underlie both kinds of functionality, but evidence for one does not establish the other. A technological role can be met at addition levels that supply a fraction of a gram per serving, whereas the health responses considered here were tested at gram-scale intakes and depend on the property surviving digestion as well as processing. Retained function refers here to the survival of such a property after formulation, processing and digestion. Studies are considered in order of increasing system complexity: bulk behavior in aqueous media, interfacial behavior in emulsions, interactions within composite food matrices, including starch-based products, flavor and sensory acceptance, and the transition from laboratory fractions to commercial preparations.

### 4.1. Solubility and Rheology

Solubility and rheology provide the first test of how preparation characteristics translate into bulk food behavior. Established hydrocolloids provide useful reference points: oat β-glucan has clearer human data connecting viscosity with metabolic outcomes, whereas xanthan gum and gum arabic serve as benchmarks for thickening and interfacial stabilization, respectively [27,90,91]. Representative EMP contrasts and their test conditions are summarized in Table 4. For EMPs, the immediate development question is preparation-specific: whether a material can disperse and generate the required viscosity within the intended matrix and processing window.

Paired preparation series reveal a recurring solubility–viscosity trade-off. In γ-irradiated *Auricularia polytricha* preparations, molecular weight decreased from 6820 to 34 kDa and apparent viscosity at approximately 10 mg/mL fell from 133 to 4.8 mPa·s [30]. Fractionation of a *Lentinula edodes* polysaccharide produced a complementary pattern: water solubility increased from 44% in P20 to approximately 97% in the most soluble subfractions, while intrinsic viscosity declined [59]. Notably, composition and molecular size changed concurrently in both series. These studies support preparation-level trade-offs between dispersion and thickening, without isolating a single structural determinant.

Concentration can shift the physical regime of the same preparation. *Auricularia polytricha* polysaccharides obtained by ultrasonic/microwave-assisted extraction behaved as Newtonian fluids at 0.1–0.5% (*w*/*v*) and developed weak-gel behavior at concentrations of 2.0% or higher [13]. A commercial *Tremella fuciformis* preparation likewise showed shear-thinning behavior under the reported emulsion conditions [14]. These thresholds define formulation windows: the required texture and product format must be specified before a working concentration can be selected.

Gastrointestinal processing adds another layer of change. During simulated digestion of a *Dictyophora indusiata* polysaccharide, apparent viscosity and dynamic moduli were broadly retained; during subsequent fecal fermentation, apparent viscosity, storage modulus and loss modulus declined while short-chain fatty acids accumulated [77]. This study coupled rheological and fermentation measurements within a single experimental framework. The initial viscosity was not maintained through the full in vitro sequence, qualifying its use in explanations of later colonic events. Matched tests in prepared foods remain scarce. Most formulated foods contain more than one phase. In emulsions, bulk viscosity is joined by interfacial adsorption and protein association.

### 4.2. Emulsifying Properties

Emulsification extends EMP functionality from a continuous aqueous phase to an oil–water interface. The reported behavior is consistent with contributions from continuous-phase viscosity, hydrophobic domains or retained proteins, and interactions with added protein emulsifiers [14,15,41,42,91]. Extraction and fractionation alter molecular-size distribution and composition, so several contributors to interfacial behavior may vary concurrently [41,59].

The *Lentinula edodes* fraction series illustrates this tension. The emulsifying activity index (EAI) decreased from 2.86 m^2^/g in P20 to 1.86 m^2^/g in P20-1 and 1.32–1.70 m^2^/g in the later fractions; the emulsion stability index (ESI) also declined slightly [59]. Fractionation yielded more compositionally resolved materials, accompanied by lower emulsifying activity under the study conditions. Molecular size and composition varied together, precluding causal attribution to purification alone.

Concentration and pH further define the usable formulation window. In palm-oil emulsions, 0.8% commercial *Tremella fuciformis* polysaccharide produced the highest reported EAI (0.98 m^2^/g) and ESI (88%); higher concentrations increased viscosity or gel-like behavior without further improvement [14]. Laboratory, crude and commercial *Tremella* preparations also displayed different EAI and ESI patterns across pH 2–10 [41]. Similarly, *Auricularia auricula* AAP-W emulsions remained uniform for 30 days at 1–3%, whereas systems containing 0.1–0.5% separated and an oil fraction of 0.7 was unstable [15]. These studies identify preparation-specific operating windows in model emulsions. The usable window may shift after heating, storage, shear or pH cycling in a finished product.

Protein association makes the system more sensitive to formulation history. Commercial *A. auricula* and *Ganoderma lucidum* polysaccharides formed emulsions with soy-protein isolate, but droplet size depended sharply on pH and the mixing ratio. For the soy-protein isolate–AAP system, mean droplet size was approximately 2 μm at pH 3 and 105 μm at pH 4 [42]. The large contrast places the functional response at the composite interface, where polysaccharide identity, protein content and electrostatic environment act together. Linked food and digestive measurements remain uncommon, although an acidified whey-protein system combined dispersion stability and microstructure with in vitro protein digestion for the same *Tremella fuciformis* polysaccharide complex [43]. *Auricularia auricula* polysaccharide and extract likewise stabilized acidified whey-protein dispersions, and heat treatment changed the complexes [44]. These food-oriented designs connect matrix performance with digestive or sensory measurements, although neither included an EMP-specific host metabolic outcome. At higher concentrations or in protein-rich foods, the same hydration and association processes also influence network formation, texture and water release.

### 4.3. Water Holding, Gelation and Protein Interaction

Water-holding, oil-holding and swelling assays provide an initial indication of how a mushroom-derived material may retain liquid during mixing or storage. *Pleurotus eryngii* insoluble dietary fiber showed a water-holding capacity of 5.16 g/g, a swelling capacity of 4.69 mL/g and an oil-holding capacity of 1.28 g/g [16]. Crude *Lentinula edodes* polysaccharides also showed extraction-route- and pH-dependent swelling [56]. These measurements are useful screening endpoints, although the fiber-rich and crude preparations contain components beyond isolated EMPs and their values remain specific to the stated hydration protocol. As concentration rises, polymer entanglement and interactions with food proteins may shift the material toward network formation. Yet critical gel concentration, gel strength and reversibility remain poorly characterized for isolated EMPs under food-relevant conditions, limiting predictions of network reinforcement, weakening or water release.

The available composite-matrix studies already show non-linear addition effects. In wheat gluten, 8% *A. auricula* polysaccharide increased the denaturation peak temperature from 52.4 to 57.2 °C and increased the enthalpy change (ΔH) from 55 to 102 J/g. At 10%, viscoelasticity and network quality declined relative to the 8% formulation [45]. A chicken–mushroom hybrid gel provides context at the whole-mushroom level: progressive replacement with *Schizophyllum commune* powder reduced hardness from 6.75 to 1.41 N and increased expressible fluid from 10.2% to 32.7% [92]. The latter response cannot be assigned specifically to EMPs, yet it exposes a practical ceiling imposed by texture and water release. An addition level that is feasible in an animal gavage model may exceed the amount that a food matrix can accommodate.

Instrumental texture defines only part of this ceiling. Hardness, rheology, differential scanning calorimetry and water release can identify mechanical limits, while sensory evaluation determines whether the resulting product remains acceptable. Finished-product studies provide direct examples. In set yogurt, 0.1% *T. fuciformis* polysaccharide improved water holding, texture and rheological stability over 21 days of cold storage [46]. A *T. fuciformis* polysaccharide–sodium caseinate system was subsequently applied in low-fat ice cream, where the selected formulation provided melt resistance [47].

Thermal analysis provides separate, narrower information. *Pleurotus eryngii* polysaccharides showed a degradation onset near 251 °C, and *L. edodes* fractions degraded mainly between 230 and 335 °C [59,93]. Different differential-scanning-calorimetry profiles were also reported for an *Auricularia cornea* polysaccharide and its zinc complex [72]. These temperatures describe behavior during analytical heating. Retention of functionality after cooking, sterilization or storage must be determined in the processed material.

### 4.4. Interaction with Starch

EMPs change how starch gelatinizes, pastes and retrogrades, and the direction of the change depends on the preparation. In sorghum starch, *Lentinula edodes* β-glucan coated the granules through electrostatic interaction and promoted retrogradation during long storage, whereas *Auricularia auricula* and *Tremella fuciformis* polysaccharides associated with starch hydrophobically and suppressed it. All three lowered the degree of gelatinization [94]. Two *Flammulina velutipes* polysaccharides from one source acted in opposite directions in rice starch. The water-extracted, α-glucan-rich preparation (103 kDa) restricted granule swelling, lowered pasting viscosity and raised the gelatinization temperature. The alkali-extracted triple-helix β-glucan (1150 kDa) increased swelling and viscosity and accelerated gelatinization. Both reduced amylose leaching and retrogradation and raised the storage and loss moduli [66]. *T. fuciformis* polysaccharide likewise raised the pasting temperature and viscosity of tiger-nut (*Cyperus esculentus*) starch and reduced amylose leaching [95]. These properties govern cooking loss, firmness and staling, so the extraction route of the polysaccharide becomes a texture variable in a starchy food.

The same associations slow starch digestion. In every model system the polysaccharide lowered in vitro hydrolysis. Two routes are involved, and both also operate in the intestinal lumen: inhibition of α-amylase and restricted enzyme access in a more viscous or more ordered matrix. A deproteinized *Tremella* preparation inhibited pancreatic α-amylase (IC50 5.76 mg/mL) and increased digesta viscosity, with negligible glucose adsorption. At 1.2 mg/mL it lowered potato-starch hydrolysis at 180 min from 91.7% to 29.7% [96]. At 0.6% in sorghum starch, *T. fuciformis* polysaccharide reduced reducing-sugar release at 20 min by 53.5% [94]. Both *F. velutipes* preparations lowered rapidly digestible starch, and the predicted glycemic index (pGI) of rice starch fell from 92.4 to 78.7 at 10–20% addition [66]. *T. fuciformis* polysaccharide raised the slowly digestible and resistant fractions of tiger-nut starch [95]. In the *F. velutipes* pair, one preparation formed a compact, hydrogen-bonded gel and the other a thick, entangled one, so a shared reduction in digestibility does not indicate a shared mechanism [66].

In finished starchy foods these effects appear within a narrow addition window, and this is where the formulation challenge lies. In corn noodles, a magnetically treated *Ganoderma lucidum* extracellular polysaccharide was tested at 0.2–1.0%, and 0.6% was the optimum. At this level cooking loss fell by 24.7%, breakage fell by 46.7% and the pGI fell from 76.4 to 72.3 [49]. In fresh wet rice noodles, the alkali-extracted *F. velutipes* polysaccharide characterized in the rice-starch model was added at 0.5–4%. Breakage was lowest at 2%, whereas resistant starch (18.9% to 22.3%) and the pGI (90.4 to 80.1) changed most at 4% [97]. In mice, the 4% noodle lowered blood glucose at 30 min by 19.7%, delayed the glucose peak to 60 min and kept plasma GLP-1 and peptide YY (PYY) elevated from 60 to 120 min; the 1% noodle did not change the glycemic response [97]. As with gluten, the response to addition level is not linear, and the level that gives the best texture is not necessarily the level that gives the largest effect on digestion. The pGI is a digestion-model value and the mouse test used a gavaged noodle suspension, so a human postprandial test of a starchy food containing a characterized EMP preparation remains to be done.

### 4.5. Flavor and Aroma

The taste and aroma for which *Flammulina velutipes*, *Pleurotus* and *Lentinula* species are valued come from low-molecular-weight compounds. Free amino acids and 5′-nucleotides give the umami taste, and eight-carbon volatiles such as 1-octen-3-ol give the characteristic aroma [98]. Ethanol precipitation, dialysis and fractionation remove most of these compounds, so the flavor that an EMP ingredient brings to a food depends on its preparation class. Whole-mushroom powders carry the flavor of the source, purified polysaccharides are close to neutral, and crude and enriched extracts lie between.

For whole-mushroom formats, flavor can be an asset or a limit, and the acceptable level is product-specific. In high-moisture extruded pea–mung bean protein, shiitake powder suppressed the beany off-notes of pea protein and improved flavor, and 20% was the sensory optimum of the 0–30% range [99]. In a pumpkin–carrot soup evaluated by 109 consumers, *Pleurotus ostreatus* powder at 2% was liked as much as the control. Liking decreased at 4% and 6%, as mushroom odor, strong taste and dark color came to describe the samples [100].

Polysaccharide preparations influence flavor indirectly through texture. In solutions thickened with a hydrocolloid, perceived flavor and sweetness intensity decreased once the polymer concentration exceeded the coil-overlap concentration, although the amount of volatiles reaching the nose was unchanged [101]. The viscosity that slows starch digestion or stabilizes an emulsion can therefore also mute taste. The EMP studies available so far used low addition levels and did not test this effect directly. Electronic-tongue measurements indicated lower bitterness in acidified whey-protein dispersions containing *Auricularia auricula* polysaccharide than in a neutral whey-protein control, although the two differed in pH [44]. At 0.1%, *Tremella fuciformis* polysaccharide caused no adverse shift in the measured flavor attributes of set yogurt during 21 days of cold storage [46]. In low-fat ice cream containing *T. fuciformis* polysaccharide–sodium caseinate emulsions, a trained panel gave the highest flavor and texture scores at 0.3%; higher levels improved melt resistance but introduced a slight powdery mouthfeel and lowered overall acceptability [47]. Electronic-tongue and electronic-nose responses describe composition, so consumer liking requires separate testing. At 0.1%, a serving of yogurt supplies a fraction of a gram of polysaccharide, well below the gram-scale intakes considered for metabolic effects. Exposure estimates should be anchored to a formulation that satisfies both technical and sensory constraints.

Color also depends on the preparation. Whole-mushroom powder markedly darkened chicken patties [12], and an alkali-extracted *Flammulina velutipes* polysaccharide produced addition-dependent color changes in wet rice noodles, which became darker and redder at higher addition levels [97]. Acceptance has already affected human testing. In the *Lentinula edodes* trial, two participants in the intervention group withdrew because they disliked the product, and no sensory evaluation had been used to adjust the formulation [86]. In the *Pleurotus ostreatus* beverage trial, participants rated the taste of the test and placebo drinks as moderate [88]. In the studies cited here, purified EMP preparations were evaluated by trained panels or instruments, and consumer acceptance was tested only for whole-mushroom formats [100].

### 4.6. Laboratory and Commercial Preparations

Preparation format can change measured performance and the relevance of that performance to real food systems. In Tremella fuciformis, laboratory TPS, crude TMS and commercial TPS-120/TPS-160 displayed distinct EAI, ESI and pH-dependent rheological profiles; molecular mass alone did not account for the pattern [41]. Commercial *A. auricula* and *G. lucidum* polysaccharides functioned in soy-protein emulsions, although the *G. lucidum* material contained measurable protein and the response depended on the protein–polysaccharide environment [42]. A direct food-application comparison also found different β-glucan contents and fat-replacement behavior for dried Pleurotus ostreatus powder and its polysaccharide extract [12]. Differences of this kind complicate comparison across preparation grades and suppliers, as well as the transfer of metabolic findings when commercial composition is incompletely reported.

Taken together, these studies support a bounded conclusion. EMP preparations can provide thickening, emulsification and matrix-modifying functions, with useful operating windows reported in selected model systems. Performance does not yet transfer reliably across extraction grades, commercial sources or food matrices. More importantly, technical and metabolic studies usually concern different preparations: metabolic investigations frequently use laboratory-prepared fractions or gavaged extracts, whereas food studies include commercial polysaccharides, crude extracts, dietary-fiber preparations and whole-mushroom matrices.

## 5. Requirements for Translation to Food Ingredients

The translation of EMP bioactivity into food ingredients can be assessed across three connected research domains. The first concerns whether the same defined preparation has been examined across processing, food-system behavior and metabolic outcomes. The second is exposure feasibility: the preparation must be deliverable at a realistic intake while retaining the property relevant to its proposed function. The third comprises preparation-specific quality, safety and regulatory evidence. These domains address different questions, and evidence in one cannot resolve uncertainty in another.

### 5.1. Linked Preparation, Function and Outcome Measurements

Bioactivity alone is insufficient for food-ingredient translation. A preparation may alter glucose or lipid endpoints in animals yet remain poorly positioned for food use if its composition, matrix behavior and feasible intake have not been examined together. Preparation identity and biological response need to be connected through food-system testing of the same defined preparation or of batches shown to meet the comparability conditions set out below (Figure 1 and Figure 2). Separate studies become translationally informative when preparation equivalence is demonstrated.

Cross-study comparison depends on how far two preparations can be treated as one material, and three situations need to be distinguished. In the first, the studies used the same preparation: material from the same source part and batch or from batches produced by the same extraction and purification sequence under the same extraction conditions. Results from such studies can be read together. In the second, the preparations were made separately, and equivalence has to be shown on four points: the same source part and preparation route, with any difference in extraction conditions stated; carbohydrate or glucan, protein, ash and phenolic contents that agree within the variation reported between batches of the preparation or, when one batch is reported, within the repeatability of the assay; molecular-weight distributions that agree when measured with the same method and calibrated against the same standards; and, where a function is claimed, a function-relevant property measured under matched conditions, such as viscosity at a stated concentration or bile-acid binding. Agreement on route alone supports the transfer of a result only as a hypothesis for testing. In the third, the route, purification depth or molecular-weight distribution differs, or the co-extracted components differ in kind; the materials are then different preparations whose results can be contrasted, as in the degradation and fractionation comparisons above, and cannot be combined. Most published pairs fall into the third situation or cannot be assigned, because molecular-weight methods differ between laboratories and protein, ash and phenolic contents are often unreported.

Translational value depends on both the breadth of measurements and the specificity of attribution. The most informative designs examine a controlled preparation or structural contrast, a quantitative food-relevant property and an in vivo or human metabolic outcome for the same material. When these elements are measured together, changes in preparation, physical behavior and outcome can be interpreted within one experiment. The responsible material property remains unresolved unless the focal variable is isolated or concurrent compositional and physicochemical changes are measured and controlled. A controlled processing contrast may support an effect of the resulting preparation even when the contribution of molecular weight, viscosity or another individual feature remains uncertain. Side-by-side extraction studies remain limited, which makes the effects of polymer structure, co-extracted components and processing conditions difficult to separate [19].

Two preparation series come closest to a linked design. The gamma-irradiated *Auricularia polytricha* series examined processing, physical behavior and a metabolic outcome together: irradiation reduced molecular weight about 200-fold and apparent viscosity about 28-fold (Table 2), and the most degraded preparation produced lower serum total cholesterol and hepatic triglyceride values than the native preparation in high-fat-diet mice [30]. This comparison supports an effect of the irradiated preparation under the tested conditions. Irradiation also changed solubility, morphology, thermal behavior and chemical features; consequently, the metabolic difference cannot be assigned specifically to molecular weight, viscosity or another single property. The alkali-extracted *Flammulina velutipes* polysaccharide series follows one characterized preparation from a rice-starch model into fresh wet rice noodles and an acute postprandial test in mice, with the water-extracted preparation from the same source serving as the contrast [66,97]. Within that series, the starch-model, noodle and postprandial results concern one preparation and can be read together, whereas the water- and alkali-extracted materials differ in route and composition and are compared as different preparations. Designs of this kind test functional delivery directly; their next step is to establish whether the characterized food and intake produce reproducible human outcomes while distinguishing the ingredient effect from changes in the surrounding recipe. Of the two, only the *Flammulina velutipes* series measured technological and health functionality for the same preparation, and its health response was limited to an acute test in mice. The irradiated series linked a property of the preparation, its viscosity, with a health response without testing the preparation in a food. A β-glucan-enriched *Lentinula edodes* mixture approaches the same design from the human side. The mixture was prepared from shiitake mushrooms by a previously described extraction route and characterized for composition. It was incorporated into commercial vegetable creams, with heating conditions and post-processing glucan content reported, and tested at a defined daily intake against placebo [86]. The trial did not establish a lipid-lowering effect. Two links also remain incomplete: the molecular-weight distribution and fine structure of the final mixture were not fully characterized, and glucan retained after heating does not show that the property relevant to the proposed lipid-lowering action was retained.

Other studies examine only part of this relationship, and the unmeasured element differs by design. *Ganoderma sinense* soluble dietary fiber and *Pleurotus eryngii* insoluble dietary fiber were characterized for hydration or holding properties and tested in animal metabolic models, but neither study included a controlled preparation contrast [16,102]. Conversely, the *Lentinula edodes* extraction comparison and fraction series linked preparation differences with techno-functional measurements and in vitro binding or enzyme-inhibition assays, without extending the comparison to an animal or human outcome [56,59]. These laboratory fractions and the β-glucan-enriched *Lentinula edodes* mixture tested in humans [86] share only their source species; their binding and inhibition results therefore cannot be carried to the trial. The *Dictyophora indusiata* digestion–fermentation study coupled rheology with short-chain fatty acid production, although both observations remained within an in vitro exposure model [77]. A *Morchella importuna* preparation was followed across holding, emulsifying and rheological measurements, simulated digestion and fecal fermentation [103]. Its wider within-preparation coverage ended before a matched host metabolic outcome. Each design addresses a specific part of the translation question. The main gap is the scarcity of studies that follow a defined preparation through processing, food-relevant function and a host metabolic outcome beyond an acute test.

### 5.2. Exposure Feasibility

Even when preparation, physical behavior and metabolic outcome are examined together, the result has limited practical value if the tested exposure cannot be reproduced in food. Animal interventions report exposure on different bases, including body-weight dose, dietary percentage, feed concentration and administered concentration. Delivery is weighted toward gavage or intragastric administration, with fewer diet-admixture designs. These approaches can test biological response, whereas food-ingredient translation additionally requires the preparation to be delivered and characterized in its intended matrix. A *Lentinula edodes* soluble dietary-fiber study, for example, incorporated the material into high-fat-diet chow at 150–450 mg/kg. This shows that diet admixture was possible in that model, although the reported denominator must be specified before the exposure can be compared across studies [53].

Representative mouse doses in Table 3 range from 50 to 800 mg/kg, although dose basis, route and duration differ across studies. Using the U.S. Food and Drug Administration (FDA) body-surface-area conversion factors (Km), these exposures correspond approximately to 0.2–3.9 g/day for a 60 kg adult [104]. This comparison locates the order of magnitude rather than a human efficacy dose. For predominantly luminal EMP actions, intestinal concentration, hydration, viscosity, binding capacity and fermentability provide the more informative exposure descriptors [27,28].

Human interventions provide more direct exposure anchors (Table 3): 750 mg/day of a *Ganoderma lucidum* polysaccharide-peptide preparation and 3–8.1 g β-glucans per day or per test meal from a *Lentinula edodes* enriched mixture and whole-mushroom *Pleurotus* and *Tremella* formats [85,86,87,88,89]. Food-system measurements provide a second anchor for delivery. A 250 mL beverage-like serving containing 1% (*w*/*v*) *Auricularia auricula* polysaccharide would supply approximately 2.5 g, and 1–3% model emulsions remained uniform during the reported 30-day test [15]. In a wheat-gluten system, an 8% addition level improved thermal and structural properties, whereas performance declined at 10% relative to the 8% formulation [45]. These examples show that gram-scale loading is physically possible in selected model matrices. They do not establish finished-product feasibility because sensory acceptance, processing history, storage stability and post-digestion function were not evaluated together.

Retained function is therefore the unresolved part of exposure feasibility. The delivered preparation must preserve viscosity, binding capacity, fermentability or another property relevant to the proposed luminal route after incorporation and processing. Purification can improve compositional definition, yet the *Lentinula edodes* fraction series showed lower emulsifying activity in the more resolved fractions than in the parent fraction [59]. Crude or enriched preparations may be easier to formulate at higher levels, although co-extracted protein, phenolics or salts reduce component-level attribution. Direct comparisons across purification depths at matched intake remain unavailable. The practical question is whether a preparation with acceptable yield and matrix compatibility can retain sufficient function without exceeding texture, viscosity or sensory limits. Thus, mass delivery appears feasible in selected systems, while functional delivery has not been established.

### 5.3. Quality, Safety and Regulatory Status

Quality, safety and regulatory conclusions apply to a defined preparation and intended use rather than to the source species alone. European novel-food consultation records illustrate this distinction. An extract powder from *Ganoderma lucidum* fruiting bodies, produced through aqueous and ultrasound-assisted extraction followed by concentration and drying, was recorded as not novel in 2025. In contrast, dehydrated *G. lucidum* mycelium powder was recorded as novel in 2019 [55]. These consultations concern different source parts and production routes. They establish material-specific novelty status rather than a species-wide conclusion on safety or health effects.

FDA generally recognized as safe (GRAS) notices show the same dependence on preparation and use. The FDA issued no-questions letters regarding notifier conclusions for β-glucans derived from *G. lucidum* mycelium at 150 mg per serving in specified food categories and for chitosan plus β-1,3-glucans from white button mushrooms at 0.015–0.15 g per 100 g of food for specified antimicrobial uses [105,106]. By contrast, evaluation of β-glucans from *Hericium erinaceus* strain BCRC 35669 was ceased at the notifier’s request in GRAS Notice (GRN) 1124 [107]. Cessation does not itself establish that the ingredient is unsafe. Likewise, a no-questions letter concerns the notified material, manufacturing process, use level and food categories; it is not a general endorsement of other preparations from the same species.

Human tolerability data are similarly bounded. In a 12-week randomized controlled trial in healthy adults, a specified *G. lucidum* β-1,3/1,6-D-glucan was administered at 200 mg/day and showed no between-group changes in liver or kidney function markers [108]. The result provides a tolerability reference for that preparation, population and dose. It cannot substitute for the safety assessment of preparations produced by different processes or intended for higher exposures.

Whole-mushroom familiarity is insufficient for evaluating a concentrated extract. Existing food-contaminant rules include maximum levels for contaminants such as cadmium and lead in relevant mushroom or fungal food categories [109]. Extraction and enrichment can change the relative amounts of polysaccharide, residual protein, ash, heavy metals, solvent residues and microbial impurities. A translation-oriented specification should address source authentication, moisture or solids basis, polysaccharide or glucan assay, protein and ash, molecular-weight profile, microbiological quality, heavy metals, relevant residual solvents, and stability under the intended storage and processing conditions. The exact dossier requirements depend on the jurisdiction, preparation and proposed use [110].

Modification routes add hazards that the source species and its unmodified extracts do not carry. Controlled degradation and chemical substitution can leave oxidant, alkali, acylating reagent, salt or enzyme residues and low-molecular-weight by-products [29,36,51,52,57]. Irradiation with γ rays at 10–1000 kGy can form radiolytic products [30]. Selenium enrichment and metal complexation introduce an element whose chemical form and contribution to total dietary intake need to be known [37,38,50,72]. The cited studies characterized structure and metabolic activity in detail, but their safety-related observations were limited to cell viability and to liver and kidney markers or histology in disease models. One study referred to earlier tolerance work [38]. Such observations record improvement in diseased animals and were not designed to detect hazards. Selenium was reported as a total content without speciation [37], and polysaccharide-stabilized selenium nanoparticles reduced HepG2 viability at 80–100 μg/mL [50]. Food-use assessment rests on residual-reagent and by-product analysis and on repeated-dose, subchronic or genotoxicity testing [110], and these lay outside the design of the studies. Until such data are available for a defined modified preparation, it stands further from GRAS or Novel Food assessment than the unmodified extracts for which notifications and a tolerability trial exist [55,105,108]. Its safety data therefore need to enter the preparation description together with structure and function.

Allergenicity requires separate consideration. Case reports document immunoglobulin E (IgE)-mediated reactions to edible mushrooms, including anaphylaxis [111]. Depending on extraction and purification, a polysaccharide-rich ingredient may retain source proteins or change their concentration relative to the whole mushroom. Residual protein should be measured and interpreted alongside source-specific allergen history, processing, intended intake and the target population [110,111]. Polysaccharide enrichment alone does not establish low allergenic potential.

Mature dietary-fiber ingredients provide a benchmark for what needs to be established for a single material. For oat and barley β-glucans and psyllium, source, intake, function-relevant properties, eligible food conditions and human lipid endpoints can be evaluated within established meta-analyses and health-claim frameworks [112,113,114,115,116,117,118,119,120]. Table 5 summarizes where current EMP studies remain short of that benchmark.

## 6. Practical Reporting and Study-Design Priorities for EMP Food-Ingredient Translation

The recurring gaps in material definition, food-system performance, exposure and biological response point toward a practical agenda for future edible mushroom polysaccharide (EMP) research. The framework begins with preparation identity and exposure then follows the preparation through formulation, processing and outcome assessment. It draws on complementary principles from nutrition-intervention reporting initiatives, the Template for Intervention Description and Replication (TIDieR), the INFOGEST static in vitro digestion protocol and the Animal Research: Reporting of In Vivo Experiments (ARRIVE) 2.0 guidelines [121,122,123,124]. It also addresses questions that arise specifically in EMP research: what material was prepared, how it was delivered, which function remained available in the food and which comparison supports the interpretation. These priorities provide a clearer basis for carrying findings from compositional analysis into food formulation and human research.

Preparation identity provides the starting point because an EMP is defined by more than its source species. Source format, such as fruiting-body or mycelial material, should be reported separately from preparation class: crude extract, polysaccharide-enriched extract, chromatographic fraction or purified polysaccharide. A useful description connects source part and batch information with the extraction sequence, purification depth, preparation class and yield basis. Basic quality information adds the composition needed to recognize the preparation across experiments: the carbohydrate or glucan assay, protein and ash contents, moisture or solids basis and an indication of batch consistency. Structural analysis can then follow the research question. Molecular-weight distribution and its analytical basis are particularly relevant when viscosity, digestion or processing stability is discussed, whereas monosaccharide composition, linkage, branching and conformation become more informative when the study proposes a structure-dependent function. This layered description keeps routine reporting manageable while preserving the features that distinguish one EMP preparation from another.

Once the preparation is recognizable, exposure places it in a biological and product context. Route, frequency, duration and dose basis define an animal intervention; diet-admixture studies also need the inclusion level, food intake and estimated consumption of the EMP itself. Human and food-matrix studies shift the emphasis to daily intake, serving size, food vehicle, timing and compliance. These quantities are most informative when the amount consumed can be read alongside the concentration used in the food and the function retained at that concentration. Mass-based animal-to-adult conversion offers one indication of scale. Proposed addition level, achievable serving size and actual intake provide the corresponding formulation anchors. Reporting them together allows a metabolic response to be considered against the amount that a realistic food can deliver.

The next step is to choose measurements that follow the intended use of the ingredient. In a beverage, development begins with dispersion, flow behavior and resistance to sedimentation or phase separation. An emulsion redirects attention to droplet stability, interfacial behavior and changes during pH adjustment, heating or storage. As the matrix becomes more structured, texture, water retention, syneresis and gel or protein-network behavior define the useful addition range.

The metabolic rationale then determines which functions should be followed beyond initial formulation. Lipid-oriented studies can combine intestinal-phase viscosity with measurements of bile-acid or cholesterol interactions. Microbiota-oriented designs instead trace resistance to upper-gastrointestinal digestion through fermentation, substrate degradation and metabolite production. Processing connects these physiological questions to the food as consumed. Heating, homogenization, pH adjustment and storage can reshape the property that motivated ingredient selection; repeating the relevant measurement after these operations shows how much of that function is retained at consumption. In a finished food, formulation quality and physical stability extend the assessment to the product level. Sensory evaluation can focus on appearance, flavor, aroma, mouthfeel, texture and overall acceptability, establishing whether the selected intake remains compatible with an edible product. Organizing the analytical panel around the intended food format and research question connects preparation choice with product performance, retained function and consumer experience.

Clear comparisons give these measurements their interpretive value. Native or parent preparations provide the natural reference for modification, degradation and purification studies, while matrix controls show whether a response arises after incorporation into food. The matching basis also needs to be explicit: equal total preparation mass, polysaccharide or glucan mass and equal functional concentration answer different questions. Experimental conditions complete the comparison. A reported value gains comparability when it is accompanied by concentration, matrix composition, pH, temperature, shear or deformation history, storage time and the principal digestion or fermentation conditions. Standardized methods such as the INFOGEST protocol offer a common starting point when gastrointestinal processing is part of the question [123]. Numerical outcomes, units and uncertainty then make the results reusable across studies. When a mechanism becomes central to the claim, pathway-appropriate perturbation can be added to the design. Taken together, these practices help researchers decide whether an EMP preparation is reproducible, deliverable, functional after processing, acceptable in food and ready for the next level of biological evaluation. Table 6 distils these considerations into proposed reporting and study-design priorities for future EMP food-ingredient research.

## 7. Limitations

While this review synthesizes current studies on edible mushroom polysaccharides and metabolic health, several important limitations should be acknowledged. First, the cited studies are an illustrative selection identified through English-language searches of two databases with targeted additions, so work published in other languages or indexed elsewhere was not covered. Second, the primary studies were not formally assessed for risk of bias, and the animal literature in particular is likely to over-represent positive findings. Third, numerical values come from single studies and were not pooled; they describe the size of an effect for one preparation in one model and do not extend to other preparations, matrices or endpoints.

## 8. Conclusions

Edible mushroom polysaccharides (EMPs) have a credible basis for development as functional food ingredients for metabolic health. Across preclinical studies, preparation-specific changes recur in glycemic control, adiposity, lipid homeostasis and hepatic lipid accumulation. Luminal assays and selected microbiota-perturbation experiments support several proposed routes. Food-system studies show that some EMP preparations can thicken aqueous systems, stabilize emulsions, retain water and modify protein networks. Yet preparations evaluated for metabolic responses are seldom followed through food processing and matrix performance. The convergence of biological activity, technological function and realistic exposure in one reproducible preparation has therefore not yet been demonstrated.

Animal dose conversions, human intervention intakes and selected food-matrix loadings overlap at gram scale. Consequently, functional delivery becomes the decisive uncertainty. A candidate ingredient must retain its relevant gastrointestinal function after formulation, processing and storage at a concentration compatible with an acceptable serving. Purification, controlled degradation and chemical modification should therefore be evaluated as preparation variables that shape matrix compatibility and biological response together.

Future progress therefore depends on studies that follow one well-characterized EMP preparation into an intended food system. Processing, storage and simulated digestion would establish whether its defining function is retained, while formulation work would determine a technically feasible and sensorially acceptable serving. Controlled comparisons in the proposed food should establish technical and sensory suitability before the same preparation advances to human testing with prespecified outcomes, justified sample sizes and endpoints matched to its proposed metabolic role. Comparisons between enriched and purified preparations at equivalent polysaccharide or glucan intake would further clarify how compositional definition and matrix compatibility influence functional delivery. Where comparable gaps separate biological testing from food-system evaluation, this preparation-centered logic may also offer a useful reference for selected fungal, cereal and plant polysaccharides.

## Figures and Tables

**Table 2:** Representative EMP preparation and modification contrasts linking preparation identity, measured response and relevance to food-ingredient development.

Preparation Contrast	Processing Variable	Measured Material Change	Study Design and Response	Food-Ingredient Implication	Ref.
Extraction-route contrast: *Lentinula edodes* crude polysaccharides	Hot-water, hot-alkaline, ultrasound-water and ultrasound-alkaline extraction	Yield range 12.9% to 35.2%; study-specific Mw not reported	Food-function and enzyme assays: swelling and inhibition varied by route	Shows that extraction route changes candidate ingredient properties; metabolic effects have not been tested in a food matrix	[56]
Depolymerization: *Auricularia auricula-judae* AAP-1 vs. DAAP-1	H_2_O_2_–ascorbic acid/succinic acid degradation	Mw 119 to 59 kDa; PDI 2.63 to 1.68	Enzyme assays and mice: lower IC_50_ values and stronger lipid/insulin responses	Matched contrast links M_w_ reduction with a stronger response; food-matrix behavior was not assessed	[29]
Process-induced depolymerization: *Flammulina velutipes* soluble dietary fiber	Steam-explosion process modification	Yield 4.64% to 8.73%; Mw 313 to 123 kDa	OA-HepG2 cells: TG suppression increased from 28.4% to 51.8%	Directly links processing to a cell response; relevance to whole-organism and food-matrix settings remains to be tested	[51]
Irradiation depolymerization: *Auricularia polytricha* APP vs. irradiated products	γ-Irradiation	Mw 6820 to 34 kDa; viscosity 133 to 4.8 mPa·s	Mice: serum TC fell from 5.93 to 4.37 mmol/L in the most degraded product	Links preparation, physical function and metabolism within one study; several irradiation-induced features still co-varied	[30]
Enzymatic-ultrasound depolymerization: *Hericium erinaceus* dietary fiber	Ultrasound-assisted enzymatic treatment	M_w_ peaks shifted downward; D_50_ particle size 314 to 177 μm	In vitro: lipase inhibition and bile-salt/cholesterol binding increased	Provides a paired in vitro link between treatment and activity; the animal arm did not compare native and treated materials	[52]
Deacetylation: native vs. deacetylated *Auricularia auricula* polysaccharides	Deacetylation	Native O-acetyl DS 0.25; O-acetyl group not detected after deacetylation	Mice: both improved NAFLD endpoints; part of the inflammatory response weakened after deacetylation	Modification effects are context-dependent; matched controls and inflammatory endpoints matter	[57]
Acetylation: *Ganoderma applanatum* GAP vs. A-GAP	Acetylation	DS 0.37; M_w_ 11.6 to 12.2 kDa	In vitro and mice: antioxidant and T2DM-related responses improved	Links substitution with activity changes; food-system behavior and matched exposure require separate evaluation	[36]
Element enrichment: *Cordyceps militaris* CMP vs. SeCMP	Biological selenium enrichment	Se content 0.10 to 5.14 mg/kg; Mw and monosaccharides not characterized	Mice: no advantage at 100 mg/kg; a stronger response occurred only at 200 mg/kg	Stronger response remains dose-confounded and cannot be assigned to enrichment alone	[37]
Metal complexation: *Auricularia cornea* ACEP vs. ACEP-Zn	Zn(II) chelation/complexation	Zn content 5.41 mg/g; apparent Mw (HPGPC) 35.5 to 32.7 kDa	Enzyme assays: α-glucosidase and α-amylase inhibition increased	Complexation changes assay behavior; regulatory and matrix implications require separate evaluation	[72]
Metal complexation: *Ganoderma lucidum* GLP vs. GLP-Cr	Cr(III) chelation	Post-complex structural characterization not repeated in the metabolic paper; unequal mass dosing	Mice: GLP-Cr generally improved glucose and lipid markers more than GLP	Activity comparison is informative but composition and dose accounting are incomplete	[38]

Notes: Values are representative anchors reported in the cited studies and are not pooled estimates. Studies were selected on the basis of a within-study comparison, verifiable numerical outcomes and a complete preparation description; entries with incomplete characterization are retained where the missing information illustrates a translational gap. AAP-1, *Auricularia auricula-judae* polysaccharide; DAAP-1, degraded AAP-1; APP, *Auricularia polytricha* polysaccharide; GAP, *Ganoderma applanatum* polysaccharide; A-GAP, acetylated GAP; CMP, *Cordyceps militaris* polysaccharide; SeCMP, selenium-enriched CMP; ACEP, *Auricularia cornea* polysaccharide; ACEP-Zn, zinc-complexed ACEP; GLP, *Ganoderma lucidum* polysaccharide; GLP-Cr, chromium-complexed GLP; D50, median particle diameter; DS, degree of substitution; HPGPC, high-performance gel-permeation chromatography; IC50, half-maximal inhibitory concentration; Mw, weight-average molecular weight; PDI, polydispersity index; TG, triglyceride; TC, total cholesterol; OA-HepG2, oleic-acid-treated HepG2 cells; NAFLD, non-alcoholic fatty liver disease; T2DM, type 2 diabetes mellitus.

**Table 3:** Representative animal and human studies of EMP-related metabolic effects.

Study Type	Model or Population	Test Material	Exposure or Study Design	Main Findings	Ref.
Animal	HFD/STZ-induced diabetic mice	*Auricularia auricula* polysaccharide AAP-M	200 mg/kg body weight for five weeks	Lower fasting and OGTT glucose than model controls	[58]
Animal	HFD/STZ-induced diabetic mice	*Cordyceps cicadae* polysaccharide CH-P	800 mg/kg	Lower OGTT AUC, HbA1c, glucose and insulin; separate microbiota/indole tests	[39]
Animal with microbiota perturbation	High-fat-diet mice	*Auricularia auricula* polysaccharides	50 or 100 mg/kg; 200 mg/kg in the perturbation study	Lower weight gain and improved metabolic indices; perturbation supported microbial participation	[32,76]
Animal	Diet-induced hepatic-steatosis mice	*Cordyceps guangdongensis* polysaccharides	400 mg/kg/day	Improved weight, OGTT AUC and lipid measures; no single mediator isolated	[40]
Human RCT	60 obese adults with cardiometabolic syndrome	*Ganoderma lucidum* polysaccharide peptide	750 mg/day (540 mg/day β-glucan) for eight weeks	No between-group differences in the primary inflammatory endpoints or in lipid or BMI outcomes	[85]
Human RCT with food delivery	57 randomized, 52 analyzed adults with untreated mild hypercholesterolemia	β-Glucan-enriched *Lentinula edodes* mixture	10.4 g/day mixture providing 3.5 g/day fungal β-glucans for eight weeks	Microbiota composition changed; primary total cholesterol, lipid and inflammatory endpoints were unchanged	[86]
Human crossover RCT with food delivery	22 adults with impaired glucose tolerance	Meal fortified with oven-dried *Pleurotus ostreatus* powder	20 g powder providing 8.1 g β-glucans; acute crossover	GLP-1, NEFA and hunger AUC changed; the primary glucose endpoint was unchanged	[87]
Human RCT with food delivery	46 adults with moderately elevated LDL-C	*Pleurotus ostreatus* powder beverage	8.4 g/day powder providing 3 g/day β-glucans for four weeks	Primary LDL-C (+0.12 mmol/L; 95% CI −0.14 to 0.34) and major lipid endpoints were unchanged; the absorption-marker signal was exploratory	[88]
Human RCT with food delivery	56 overweight/obese adults with prediabetes (28/28)	*Tremella fuciformis* beverage (boiled whole-mushroom extract, 15% *w*/*v*)	180 mL once daily for 12 weeks; declared 6.4 g β-glucan per serving	Within-group decreases in HbA1c (6.03% to 5.96%) and waist circumference (95.2 to 93.5 cm); glucose tolerance, insulin indices and lipids unchanged; exploratory design	[89]

Notes: Animal studies were selected to illustrate differences in preparation, exposure and design; the human rows list all controlled trials that met the inclusion rule. AUC, area under the curve; BMI, body mass index; CI, confidence interval; GLP-1, glucagon-like peptide-1; HbA1c, glycated hemoglobin; HFD, high-fat diet; LDL-C, low-density lipoprotein cholesterol; NEFA, non-esterified fatty acids; OGTT, oral glucose tolerance test; RCT, randomized controlled trial; STZ, streptozotocin. AAP-M and CH-P are study-specific preparation codes.

**Table 4:** Representative preparation- and system-dependent techno-functional behaviors of edible mushroom polysaccharides.

Functional Focus	Tested Preparation	Test System and Key Condition	Key Finding and Formulation Relevance	Ref.
Molecular size and rheology	Irradiated *A. polytricha* APP/RAPP	Aqueous dispersions; four irradiation levels	M_w_ decreased from 6820 to 34 kDa; viscosity decreased from 133 to 4.8 mPa·s. Thickening weakened across the series.	[30]
Fraction-dependent functionality	*L. edodes* P20 and fractions	Solubility and emulsion assays	Solubility increased from 44% to approximately 97%; EAI decreased from 2.86 to 1.32–1.70 m^2^/g.	[59]
Concentration-dependent rheology	UMAE-derived *A. polytricha* polysaccharide	0.1–3.0% aqueous solutions; 25 °C	Newtonian at 0.1–0.5%; weak gel at ≥2.0%. Concentration changed the physical regime.	[13]
Emulsification	Commercial *T. fuciformis* polysaccharide	Palm-oil O/W emulsions; TFP and oil varied	At 0.8%, EAI was 0.98 m^2^/g and ESI was 88%; higher levels became overly viscous or gel-like.	[14]
Emulsion stability	*A. auricula* AAP-W	O/W emulsions; 0.1–3%; 30-day storage	Systems containing 1–3% remained uniform; those containing 0.1–0.5% separated.	[15]
Preparation format	Laboratory, crude and commercial *T. fuciformis* preparations	Emulsion assays across pH 2–10	EAI and ESI profiles differed by preparation; laboratory TPS had the highest EAI and TPS-160 the highest ESI.	[41]
Protein-assisted emulsification	Commercial AAP or GLP with soy-protein isolate	pH and mixing ratio varied	SPI–AAP droplets were approximately 2 μm at pH 3 and 105 μm at pH 4, showing strong condition dependence.	[42]
Gluten interaction	*A. auricula* polysaccharide	Wheat gluten with 0–10% AAP	The response peaked at 8%; network performance weakened at 10%, defining a non-linear addition window.	[45]
Starch interaction	Water- and alkali-extracted *F. velutipes* polysaccharides (103 and 1150 kDa) with rice starch	5–20% addition; pasting, retrogradation and in vitro digestion	Opposite effects on swelling and pasting viscosity; both reduced amylose leaching, retrogradation and RDS; pGI 92.4 to 78.7 at 10–20%.	[66]
Starch interaction in a finished food	Magnetically treated *G. lucidum* extracellular polysaccharide in corn noodles	0.2–1.0% addition; cooking quality, texture and in vitro digestion	0.6% was optimal: cooking loss −24.7%, breakage −46.7%; pGI 76.4 to 72.3.	[49]

Notes: Values are study-specific and are not intended for cross-study ranking. Examples were selected where the preparation, test conditions and numerical food-system outcomes were reported. AAP, *Auricularia auricula* polysaccharide; APP, *Auricularia polytricha* polysaccharide; EAI, emulsifying activity index; ESI, emulsion stability index; GLP, *Ganoderma lucidum* polysaccharide; Mw, weight-average molecular weight; O/W, oil-in-water; RAPP, irradiated APP; SPI, soy-protein isolate; TFP, *Tremella fuciformis* polysaccharide; TPS, *T. fuciformis* polysaccharide; UMAE, ultrasonic/microwave-assisted extraction; RDS, rapidly digestible starch; pGI, predicted glycemic index from in vitro digestion. AAP-W, P20, TPS-120 and TPS-160 are study-specific preparation codes.

**Table 5:** Key gaps between edible mushroom polysaccharides and mature dietary-fiber ingredient examples.

Evaluation Dimension	Current EMP Studies	Mature Dietary-Fiber Comparator	Main Gap for Translation
Source and material specification	Studies include purified fractions, enriched extracts, dietary-fiber preparations, commercial polysaccharides and whole-mushroom matrices, with uneven characterization.	Eligible oat/barley β-glucan and psyllium sources, assays and serving contributions are specified in established claim frameworks [112,114,115,116].	EMP material classes, compositional thresholds and assay specifications are not harmonized for claim-oriented comparison.
Function-relevant properties	Molecular weight, viscosity, binding and fermentation are measured unevenly and seldom in the preparations used for efficacy testing.	Human oat β-glucan studies link dose and physicochemical behavior with LDL-C response [117].	Reported EMP mass cannot yet be interpreted consistently as a functionally active intake.
Food-matrix dependence	Selected preparations alter emulsions, gels and protein matrices, but function after processing, storage and digestion is rarely connected with metabolic testing.	Oat β-glucan lipid responses can vary with the delivery matrix [118].	Retention of the relevant EMP property in finished foods remains poorly resolved.
Human endpoint support	Controlled studies use different preparation classes, populations and endpoints, with limited replication [85,86,87,88,89].	Meta-analyses support LDL-C reduction for oat β-glucan and psyllium under defined intake conditions [119,120].	Repeated human evidence is lacking for a consistent EMP preparation class and intake basis.
Intake and serving size	Animal mass-scale estimates, observed human intakes and selected matrix calculations overlap at gram scale, but retained function is unresolved.	Daily intake and per-serving contributions are defined for several soluble-fiber claim examples [112,114,115,116].	Preparation-specific intake targets are not linked to retained function in the intended food matrix.
Regulatory status and claim substantiation	Novel Food and GRAS examples apply to individual preparations and intended uses [55,105,106,107].	Established fiber examples connect source, amount, eligible food conditions and claim wording [112,113].	Existing material-specific decisions cannot be generalized across EMP species, source parts or extraction routes.

Notes: Entries summarize the cited EMP studies and established dietary-fiber examples according to the requirements for food-ingredient development. LDL-C, low-density lipoprotein cholesterol.

**Table 6:** Practical reporting and study-design recommendations for future EMP food-ingredient research.

Priority Area	Core Information	Context-Specific Information	Decision Supported
Source and preparation identity	Species; source part; cultivation or commercial source; batch or catalog information	Strain, cultivar or authentication details when source variation is central	Whether the tested material can be recognized and reproduced and whether two studies used the same source part, route and extraction conditions
Preparation and basic quality	Extraction and purification sequence; material class; yield basis; carbohydrate or glucan assay; protein, ash and moisture or solids basis	Batch comparison and process-relevant residual components, including residual reagents, by-products and element form for modified preparations	Whether separately prepared batches agree closely enough in composition to be treated as one preparation or must be compared as different preparations
Structural characterization	Molecular-weight method, calibrant and reported distribution; monosaccharide profile	Linkage, branching, conformation or other descriptors linked to the study hypothesis	Whether molecular-weight distributions obtained by the same method agree between preparations and which structural features can be related to processing, function or response
Exposure and product context	Dose basis; route; frequency; duration; food vehicle; actual intake where available	Serving size, addition level, meal timing, background diet and compliance	Whether the tested exposure can be delivered in the proposed food format
Food-system performance	Matrix composition, EMP concentration and the principal processing conditions	Dispersion, rheology, emulsion stability, gelation, texture, water retention or syneresis according to product type	Whether the preparation performs within a useful formulation window
Retained function and stability	Relevant processing steps and storage conditions; post-process characterization	Retained viscosity, binding, emulsification, network behavior, digestibility or fermentability	Whether the property of interest survives processing, storage and delivery and whether it agrees between preparations under matched conditions
Finished-product quality and sensory response	For formulated foods: recipe, EMP addition level, appearance and physical stability	Color, taste, aroma, mouthfeel, texture, overall acceptability and storage-related sensory change; panel or consumer method and evaluation conditions	Whether the proposed intake is compatible with product quality and consumption
Comparators and outcome reporting	Native, parent or matrix control; matching basis; numerical outcomes with units and uncertainty	Pathway-appropriate perturbation or mediation design when a mechanistic claim is pursued	Whether preparation, dose, matrix and mechanism effects can be distinguished

Notes: Recommendations are derived from the research gaps discussed in this review and from the cited reporting frameworks [121,122,123,124].

## Data Availability

No new data were created or analyzed in this study. Data sharing is not applicable to this article.

## Supplementary Materials

The following supporting information can be downloaded at https://www.mdpi.com/article/10.3390/foods15193514/s1, Table S1: Web of Science and PubMed search strategies; Table S2: Eligibility criteria applied to the candidate publications.

## Abbreviations

The following standard abbreviations are used in this manuscript. Study-specific sample and polysaccharide-fraction codes follow the original publications and are defined at first mention in the text and table footnotes rather than listed here.

AAP	*Auricularia auricula* polysaccharide
APP	*Auricularia polytricha* polysaccharide
ARRIVE	Animal Research: Reporting of In Vivo Experiments
AUC	area under the curve
BMI	body mass index
D_50_	median particle diameter
DES	deep eutectic solvent
DS	degree of substitution
EAI	emulsifying activity index
EMP	edible mushroom polysaccharide
ESI	emulsion stability index
FDA	U.S. Food and Drug Administration
FMT	fecal microbiota transplantation
GLP	*Ganoderma lucidum* polysaccharide
GLP-1	glucagon-like peptide-1
GRAS	generally recognized as safe
GRN	GRAS notice
HbA1c	glycated hemoglobin
HFD	high-fat diet
HPGPC	high-performance gel-permeation chromatography
hsCRP	high-sensitivity C-reactive protein
IC_50_	half-maximal inhibitory concentration
IgE	immunoglobulin E
IL-6	interleukin-6
INFOGEST	standardized static in vitro digestion protocol developed by the INFOGEST network
K_m_	body-surface-area dose conversion factor
LDL-C	low-density lipoprotein cholesterol
M_w_	weight-average molecular weight
NaDES	natural deep eutectic solvent
NAFLD	non-alcoholic fatty liver disease
NEFA	non-esterified fatty acids
OA	oleic acid
OGTT	oral glucose tolerance test
PDI	polydispersity index
pGI	predicted glycemic index
PYY	peptide YY
RCT	randomized controlled trial
RDS	rapidly digestible starch
SCFA	short-chain fatty acid
SPI	soy protein isolate
STZ	streptozotocin
T2DM	type 2 diabetes mellitus
TC	total cholesterol
TFP	*Tremella fuciformis* polysaccharide
TG	triglyceride
TIDieR	Template for Intervention Description and Replication
TNF-α	tumor necrosis factor-α
ΔH	enthalpy change
