# Supplementary material for: Edible Mushroom Polysaccharides as Functional Food Ingredients for Metabolic Health: Preparation, Techno-Functional Behavior, and Requirements for Translation
Source: Foods. 2026 Oct 1;15(19):3514. doi: 10.3390/foods15193514 (PMC13664489; doi:10.3390/foods15193514)
Supplement: Supplementary file 1 [file foods-15-03514-s001.zip › foods-4570597-supplementary.pdf]

Supplementary Materials

Title: Edible Mushroom Polysaccharides as Functional Food Ingredients for Metabolic Health: Preparation, Techno-Functional Behavior, and Requirements for Translation

S1. Literature Search

The primary studies considered in the review were identified through structured searches of the Web of Science Core Collection (Science Citation Index Expanded and Social Sciences Citation Index) and PubMed for English-language articles published from 2021 to 2026. Each search combined three concept blocks with the Boolean operator AND: fungal source, polysaccharide material and metabolic outcome; synonyms within a block were combined with OR. The Topic field was searched in Web of Science (title, abstract, author keywords and Keywords Plus) and the Title/Abstract field in PubMed. The complete strings are reproduced in Table S1 as run. Records from the two databases were combined and duplicates were removed by DOI, title and author before screening, leaving 222 candidate publications.

Table S1. Web of Science and PubMed search strategies.

Database and field	Search string (as run)	Filters and period
Web of Science Core Collection (SCIE, SSCI); Topic (TS) field	**Source block:** TS=("Edible mushroom*" OR "Medicinal fungi*" OR Basidiomycota OR Agaricales OR Ganoderma OR Lentinula OR Grifola OR Pleurotus OR Cordyceps OR Auricularia OR Hericium OR Inonotus OR Phellinus OR Agaricus OR Dictyophora OR Ascomycota)<br>**Material block:** AND TS=("Polysaccharid*" OR "Beta-glucan*" OR "Alpha-glucan*" OR "Heteroglycan*" OR "Proteoglycan*" OR Lentinan OR "Dictyophora indusiata polysaccharide" OR "β-glucan*" OR "α-glucan*" OR "alpha-glucan" OR "beta-glucan*")<br>**Outcome block:** AND TS=(("Metabolic Diseases" OR "Metabolic Syndrome") OR ("Diabetes Mellitus, Type 2" OR "type 2 diabetes" OR Obesity OR "Insulin Resistance") OR ("Dyslipidemia*" OR "Hyperlipidemia*") OR ("fatty liver" OR "Non-alcoholic Fatty Liver Disease" OR "Metabolic dysfunction-associated fatty liver disease" OR MAFLD OR "Hepatic Steatosis" OR NAFLD) OR ("Diabetic Cardiomyopathy" OR Atherosclerosis) OR ("Glucose Intolerance" OR "Diabetic Dyslipidemia"))	Publication years 2021–2026; document type Article; language English; early-access records retained

PubMed; Title/Abstract field	**Source block:** ("edible mushroom"[Title/Abstract] OR "medicinal fungi"[Title/Abstract] OR "Basidiomycota"[Title/Abstract] OR "Agaricales"[Title/Abstract] OR "Ganoderma"[Title/Abstract] OR "Lentinula"[Title/Abstract] OR "Grifola"[Title/Abstract] OR "Pleurotus"[Title/Abstract] OR "Cordyceps"[Title/Abstract] OR "Auricularia"[Title/Abstract] OR "Hericium"[Title/Abstract] OR "Inonotus"[Title/Abstract] OR "Phellinus"[Title/Abstract] OR "Agaricus"[Title/Abstract] OR "Dictyophora"[Title/Abstract] OR "Ascomycota"[Title/Abstract])<br>**Material block:** AND (polysaccharid*[Title/Abstract] OR beta-glucan*[Title/Abstract] OR alpha-glucan*[Title/Abstract] OR heteroglycan*[Title/Abstract] OR proteoglycan*[Title/Abstract] OR lentinan[Title/Abstract] OR "Dictyophora indusiata polysaccharide"[Title/Abstract] OR β-glucan*[Title/Abstract] OR α-glucan*[Title/Abstract])<br>**Outcome block:** AND ("Metabolic Diseases"[Title/Abstract] OR "Metabolic Syndrome"[Title/Abstract] OR "Diabetes Mellitus, Type 2"[Title/Abstract] OR "type 2 diabetes"[Title/Abstract] OR Obesity[Title/Abstract] OR "Insulin Resistance"[Title/Abstract] OR Dyslipidemia*[Title/Abstract] OR Hyperlipidemia*[Title/Abstract] OR "fatty liver"[Title/Abstract] OR "Non-alcoholic Fatty Liver Disease"[Title/Abstract] OR "Metabolic dysfunction-associated fatty liver disease"[Title/Abstract] OR MAFLD[Title/Abstract] OR "Hepatic Steatosis"[Title/Abstract] OR NAFLD[Title/Abstract] OR "Diabetic Cardiomyopathy"[Title/Abstract] OR Atherosclerosis[Title/Abstract] OR "Glucose Intolerance"[Title/Abstract] OR "Diabetic Dyslipidemia"[Title/Abstract])<br>**Date clause:** (2021-01-01[Date - Publication] : 2026-08-11[Date - Publication])	Publication date 2021-01-01 to 2026-08-11; language English

Note: The Greek-letter and transliterated spellings of β-glucan and α-glucan were both included because indexing varies between records.

S2. Eligibility Criteria and Screening

The 222 candidate publications were screened in two stages, by title and abstract and then by full text, independently by two authors, with disagreements resolved by discussion. Each publication was assessed against the four criteria in Table S2 and was retained when all four were met; 146 primary studies were retained and 76 were excluded. Human trials, food-system and extraction studies, regulatory documents and reporting guidelines cited in the review were identified through targeted searches and reference lists and were not required to fall within the 2021–2026 period.

Table S2. Eligibility criteria applied to the candidate publications.

Criterion	Retained	Excluded
Material	Identifiable fungal polysaccharide, glucan-rich fraction, polysaccharide peptide, modified polysaccharide or mushroom preparation with a measured polysaccharide basis	Non-fungal material; mushroom material without a stated or measured polysaccharide basis
Attribution	Defined preparation with an interpretable comparator; multicomponent material whose composition allowed the contribution of the fungal preparation to be described	Mixture or combined intervention in which the fungal polysaccharide-containing preparation could not be identified or its contribution described
Outcome	Glucose regulation, insulin resistance, adiposity or body weight, lipid metabolism, hepatic lipid accumulation, or a mechanistic or food-relevant outcome for the preparation	No metabolic, mechanistic or food-relevant outcome
Publication type	Primary research article reporting original data	Corrigendum, editorial, conference abstract or other non-primary publication

Note: Studies retained on a food-relevant or mechanistic outcome alone were used for the preparation, food-system and mechanism sections and were not treated as metabolic outcome studies. Controlled human studies were included when the tested product was a defined EMP preparation or a mushroom food format with a stated polysaccharide or β-glucan basis and a metabolic endpoint was reported.
